# The Food Additive β-Caryophyllene Exerts Its Neuroprotective Effects Through the JAK2-STAT3-BACE1 Pathway

**DOI:** 10.3389/fnagi.2022.814432

**Published:** 2022-02-28

**Authors:** Yujia Zhang, Qiaoyan Huang, Sichen Wang, Ziqian Liao, Haichao Jin, Shuo Huang, Xiao Hong, Yiming Liu, Jie Pang, Qing Shen, Qingcheng Wang, Changyu Li, Liting Ji

**Affiliations:** ^1^School of Pharmaceutical Sciences, Zhejiang Chinese Medical University, Hangzhou, China; ^2^The First School of Clinical Medicine, Zhejiang Chinese Medical University, Hangzhou, China; ^3^Department of Cardiology, Zhejiang Provincial People’s Hospital, Affiliated People’s Hospital of Hangzhou Medical College, Hangzhou, China; ^4^Collaborative Innovation Center of Seafood Deep Processing, Zhejiang Province Joint Key Laboratory of Aquatic Products Processing, Institute of Seafood, Zhejiang Gongshang University, Hangzhou, China; ^5^Department of Cardiology, Hangzhou Linping Hospital of Traditional Chinese Medicine, Linping, China; ^6^Academy of Chinese Medical Sciences, Zhejiang Chinese Medical University, Hangzhou, China

**Keywords:** Alzheimer’s disease, amyloid-β protein precursor, amyloid β, PC-12 cell, β-caryophyllene, JAK2-STAT3-BACE1

## Abstract

Despite extensive research on Alzheimer’s disease (AD), its diagnosis and treatment remain challenging, and no effective therapies are currently available. Amyloid β (Aβ) extracellular plaques and intracellular neurofibrillary tangles are the histological characteristics of AD that have been directly linked to neuropathological events such as synaptic and neuronal cell loss. In this study, we explored whether the “JAK2-STAT3-BACE1” pathway is involved in neuroprotection conferred by the food flavouring agent β-caryophyllene (BCP). PC-12 cells with overexpressed amyloid-β protein precursor (APP) were utilised to construct an AD model *in vitro*, which was then split into four groups, namely control, empty vector, APP overexpression, and BCP (5, 10, and 20 μM). CCK-8 was used to evaluate cell viability, immunofluorescence was utilised to examine synaptic morphology, and quantitative real-time polymerase chain reaction and western blot were used to examine gene and protein expression levels. The relative expression levels of JAK2, STAT3, and BACE1 mRNA in the transfected PC-12 cells were found to be significantly upregulated. The cell morphology altered dramatically 72 h after transfection, becoming rounder, with a decrease in cell number. BCP exhibited the potential to dramatically increase PC-12 cell viability while protecting cell morphology. BCP inhibited APP, JAK2, STAT3, BACE1 mRNA and BACE1 protein overexpression, as well as JAK2 and STAT3 hyperphosphorylation. Molecular docking simulated the docking of BCP with JAK2, STAT3, BACE1, CB2. And JAK2 was found to be the most stable protein. In conclusion, inhibition of the “JAK2-STAT3-BACE1” signalling pathway may be one of the mechanisms through which BCP protects neurons and antagonises Aβ’s neurotoxicity.

## Introduction

Currently, approximately 50 million people worldwide suffer from dementia, and the majority of these people have Alzheimer’s disease (AD). AD is a neurodegenerative disease whose major risk factor is age (Nguyen and Endres, 2021). Despite decades of research in this field, diagnosing AD remains challenging, and no effective therapies are available to date. According to a recent pathological study, patients with AD exhibit neuropathological features such as synaptic dysfunction, neuron loss, an inflammatory microglial response to dying cells, and brain microvasculature damage. Amyloid β (Aβ) extracellular plaques and intracellular neurofibrillary tangles are the histopathological characteristics of AD that have been directly linked to neuropathological events such as synaptic and neuronal cell death (Cisternas et al., 2020; Fracassi et al., 2020). Aβ oligomers can exert their toxic effect through multiple mechanisms by interacting with the synapses (Zolochevska and Taglialatela, 2020). For instance, a study found severe AD symptoms such as a defect in basal neurotransmission and the spatial memory damage in tau-knockout and amyloid precursor protein (APP) overexpression mice (Puzzo et al., 2020). In addition, the mice with reduced Aβ plaque load in the hippocampus performed significantly better in cognitive tests (Cone et al., 2021). Many studies have proved that Aβ exerts a significant impact on AD onset and progression, although drugs to combat this mechanism are still lacking.

β-Caryophyllene (BCP) is a spicy, peppery terpene found in various spices and edible plants, and it has a dry and sweet flavour. It has been approved by the Food and Drug Administration as a ‘generally recognised as safe’ food additive or ingredient in cosmetics due to its distinct flavour and noteworthy safety profile (FDA, 2020). According to research, BCP confers neuroprotection in several animal models of cognitive impairment. Yang et al. (2017) found that BCP can reduce the cerebral infarction volume, cerebral oedema, and neurological deficits in mice, in addition to exerting neuroprotective effects. Hu et al. (2017) found that BCP can inhibit neuroinflammation caused by Aβ oligomers in BV-2 microglia. Ojha et al. (2016) discovered that BCP inhibits proinflammatory cytokines and inflammatory mediators such as COX-2 and iNOS. However, the mechanism of its neuroprotective action remains to be investigated further.

Recently, our group found that BCP may protect SH-SY5Y cells against Aβ treatment, while inhibiting JAK2 expression (Zhang et al., 2020). BACE1, an essential protein in APP synthesis, can be activated by the “JAK2-STAT3” pathway (Bera et al., 2020). Therefore, BCP’s neuroprotective effect may be related to the “JAK2-STAT3-BACE1” pathway. We studied the effects of BCP on neuronal injury caused by APP overexpression and excessive activation of “JAK2-STAT3-BACE1” *in vitro* to provide preliminary data for the neuroprotective effect of BCP and drug design.

## Materials and Methods

### Cell Culture

Highly differentiated rat pheochromocytoma cells (PC-12) purchased from the Center for Excellence in Molecular Cell Science, CAS, were grown in Dulbecco’s modified Eagle’s medium (C11995500BT, Gibco, New York, NY, United States), containing 10% foetal bovine serum (1966174C, Gibco, New York, NY, United States) and 1% penicillin streptomycin (15140-122, Gibco, New York, NY, United States), and incubated at 37°C under 5% CO_2_ atmosphere. Cell medium was replaced every 2 days, and the cells were sub-cultured once they reached 80% confluence. The cells were divided into four groups: control group (CN) without special treatment; empty vector group (EVG), with PC-12 cells transfected with empty vectors plasmid; overexpression group (OE), with PC-12 cells transfected with human APP plasmid; and BCP, with PC-12 cells transfected for 48 h and incubated with different concentrations of BCP (5, 10, and 20 μM) for 24 h.

### Transient Transfections

According to the manufacturer’s instructions, 2.5 μg of human APP plasmid (500 ng/μl, GCPE0196596, Shanghai Genechem, Shanghai, China) or empty vector plasmid (3894 ng/μl, P18111400, Shanghai Genechem, Shanghai, China) was mixed with Lipofectamine 3000 (L3000008, Invitrogen, Carlsbad, CA, United States) in Opti-MEM I (31985062, Gibco, New York, NY, United States) and incubated for 15 min at room temperature before transfection in 1 mL of the culture medium for 48 h.

### Viability Assay of PC-12 Cells

The viability of PC-12 cells was determined using the Cell Counting Kit-8 (CCK-8, HY-K0301-500T, Dojindo, Japan). According to the specifications, CCK8 reagent was added to the cells, and the cells were incubated for 1 h after treating the cells as required. Cell death/proliferation was evaluated using a configurable multi-mode microplate reader (BioTek Synergy H1, Santa Clara, CA, United States) to measure the optical density values.

### Observation of Neurite Morphology of PC-12 Cells by Immunofluorescence

The cells were seeded in a 24-well plate with poly-L-lysine glass slides and cultured in an incubator for 24 h, after which the medium was removed, and the cells were washed with phosphate-buffered saline (PBS, No. P1010, Solarbio, Beijing, China). The cells were then sequentially treated with 4% paraformaldehyde (C11325432, Macklin, Shanghai, China), 0.5% Triton X-100 (MKBQ0896V, Sigma-Aldrich, St. Louis, MO, United States), and 5% BSA (A8020, Solarbio, Beijing, China). The cells were gently washed with PBS each time when the reagent was changed. The treated cells were incubated with β-actin (13E5) Rabbit mAb (4970, CST, MA, United States) overnight. The antibody was recovered, washed with PBS, and then incubated with goat anti-rabbit IgG (RS23220, Immunoway, Shanghai, China) mixed with DAPI (097M4085V, Sigma, St. Louis, MO, United States) for 2 h in the dark. After washing with PBS, the slides were removed, placed on the slides with anti-fluorescence attenuation mounting tablets (10142592, Dako, Copenhagen, Denmark), and observed under a fluorescence inverted microscope.

### Detection of Gene Expression Through Quantitative Real-Time PCR

We strictly followed the RNA Extraction Kit (AJ31039A, TaKaRa, Japan) instructions for total cell RNA extraction. We prepared a 30 μl reverse transcription system according to the TaKaRa Reverse Transcription Kit (AK22355A, TaKaRa, Japan) and the TaKaRa Fluorescence Quantitative Kit (AI71774A, TaKaRa, Japan) instructions. The transcription reaction conditions were as follows: 37°C, 15 min; 85°C, 5 s. Real-time PCR reactions were carried out in the CFX96 Real-Time System (BIO-RAD, CA, United States) by using TB green™ Premix Ex Taq™ II (Tli RNaseH Plus) (RR820A, TaKaRa, Japan). Cycling conditions were: 95°C for 30 s, followed by 40 cycles at 95°C for 5 s, 60°C for 30 s, and 72°C for 30 s. Gene expression was quantified using the comparative cycle threshold method (ΔΔCT). SANGON biotech (Shanghai, China) designed and synthesised the primer used, and the primer sequence is given in Table 1.

**TABLE 1:** Primer sequences used in the study.

Gene	Primer Sequence (5′ -3′)
JAK2-F	AAGTGCGTGCGAGCGAAGATC
JAK2-R	ACTGCTGAATGAACCTGCGGAATC
STAT3-F	AGGGCTTCTCGTTCTGGGTCTG
STAT3-R	CTCCCGCTCCTTGCTGATGAAAC
BACE1-F	GTCCTTCCGCATCACCATCCTTC
BACE1-R	ACTGTGAGACGGCGAACTTGTAAC
GAPDH-F	GACATGCCGCCTGGAGAAAC
GSPDH-R	AGCCCAGGATGCCCTTTAGT
APP-F	AGGACTGACCACTCGACCAG
APP-R	CGGGGGTCTAGTTCTGCAT

### Analysis of JAK2, STAT3, and BACE1 Peptide Levels by Western Blotting

The quantities of JAK2, STAT3, and BACE1 were estimated through western blotting. After removing the medium, the cells were rinsed with PBS two times. Then, total proteins from cultured cells were extracted with RIPA lysis (P0013C, Beyotime, Shanghai, China) buffer mixed with protease inhibitors (CW2200, CWBIO, Beijing, China), phenylmethanesulfonyl fluoride (ST506, Beyotime, Shanghai, China) and Phosphatase Inhibitor Cocktail (CW2383, CWBIO, Beijing, China). The protein concentrations in the cell lysates were determined using a BCA protein assay kit (P0012, Beyotime, Shanghai, China). Then 20 μg of the extracted protein was subjected to 10% SDS-PAGE. The blots were transferred onto the polyvinylidene fluoride membranes (1620177, BIO-RAD, CA, United States) and blocked for 2 h with 10% non-fat milk blocking buffer. Then, the blots were incubated overnight at 4°C with purified anti-β-amyloid, 1-16 antibody (1:1000, #SIG-39320, Biolegend, CA, United States), JAK2 antibody (1:1000, AF6022, Affinity, Jiangsu, China), phospho-JAK2 (tyr1007) antibody (1:1000, AF3022, Affinity, Jiangsu, China), phospho-STAT3 (tyr705) antibody (1:1000, AF3293, Affinity, Jiangsu, China), STAT3 antibody-c-terminal (1:1000, AF6294, Affinity, Jiangsu, China), anti-GAPDH antibody (1:5000, ET1601-4, HuaAn, Shandong, China) or rabbit anti-BACE1 polyclonal antibody (1:2000, bs0164r, BIOSS, Beijing, China). After incubation with horse radish peroxidase (HRP)-conjugated second antibodies, the blots were visualised using the ECL reagent (#KF005, Affinity, Jiangsu, China) under standard conditions and quantified using the ChemiDoc Touch System (BIO-RAD, CA, United States). After stripping, the membranes were re-probed for GAPDH, which was used as a loading control.

### Molecular Docking

Molecular docking study was performed to investigate the binding mode between the compound and proteins by using Autodock vina 1.1.2 (Trott and Olson, 2010). The three-dimensional (3D) structure of the proteins was downloaded from Pubchem (pubchem.ncbi.nlm.nih.gov). The 2D structure of the compound was drawn using ChemBioDraw Ultra 14.0 and then converted to the corresponding 3D structure by using ChemBio3D Ultra 14.0 software. The AutoDockTools 1.5.6 package (Sanner, 1999; Morris et al., 2009) was employed to generate the docking input files. The ligand was prepared for docking by merging the non-polar hydrogen atoms and defining rotatable bonds. For Vina docking, the default parameters were used, if not mentioned otherwise. The best-scoring pose judged by the Vina docking score was selected and visually analysed using PyMoL 1.7.6 software.^1^

### Statistical Analysis

Statistical significance of the experiments involving two groups was assessed using the Student’s *t*-test. One-way ANOVA was employed for determining differences in the mean values among multiple groups. For all statistical analyses, GraphPad Prism 5.04 software was used.

## Results

### Amyloid-β Protein Precursor Overexpression Induced Apoptosis of PC-12 Cells

**FIGURE 1:**
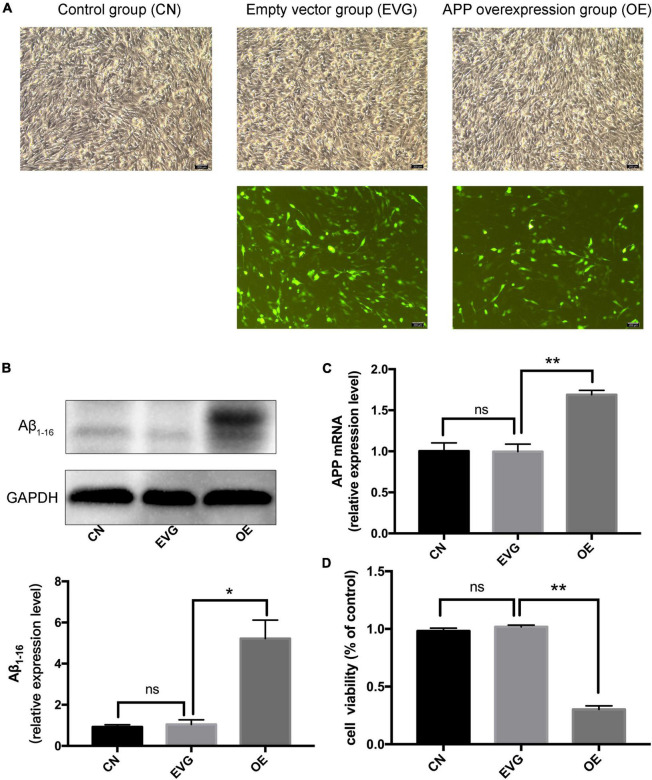
The expression of APP and viability of PC-12 cells after transfection. CN denotes control group without special treatment; EVG denotes the empty vector group in which the PC-12 cells were transfected with empty vectors plasmid; while OE denotes the group in which the PC-12 cells were transfected with human APP plasmid. **(A)** Observation of the morphology of PC-12 cells transfected for 48 h by using a fluorescence inverted microscope. **(B,C)** The expression level of Aβ_1–16_ protein and APP mRNA in PC-12 cells 48 h after transfection. **(D)** Cell viability of PC-12 cells 48 h after transfection. Compared with CN, *^ns^ p* > 0.05. Compared with EVG, *p < 0.05, **p < 0.01. One-way ANOVA with Bonferroni’s *post hoc* test. Scale bar = 200 μm.

After 48 h of transfection, the PC-12 cells displayed a high fluorescence expression and a normal cell state (Figure 1A); however, the PC-12 cells with APP gene overexpression displayed more Aβ and low cell viability. Since the antibody Aβ_1–16_ recognises the Aβ_1–42_ protofibril (Colvin et al., 2017), we selected this antibody for western blot detection. No difference was observed in the APP mRNA and protein levels between the EVG group and the control group. We discovered that the mRNA and protein levels of APP were significantly higher in PC-12 cells of the OE group than in those of the EVG group (Figures 1B,C). Cell viability of the OE group was significantly lower than that of the EVG group 72 h after transfection, as measured by CCK-8 (Figure 1D). These results indicated that the nerve cell injury model caused by APP gene overexpression was effectively constructed.

### β-Caryophyllene Attenuated PC-12 Cell Apoptosis in the Amyloid-β Protein Precursor Overexpression Group

**FIGURE 2:**
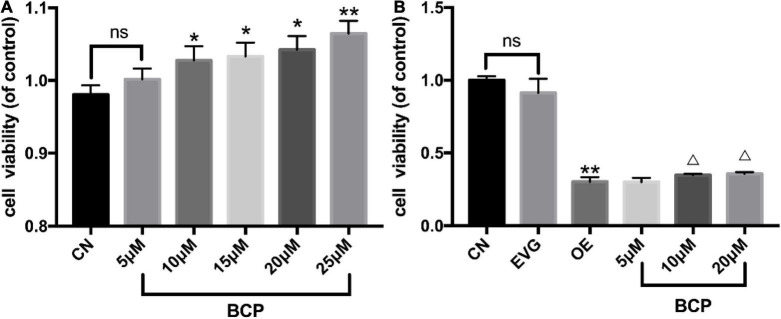
Effect of different doses of BCP on the viability of PC-12 cells. CN denotes the control group without special treatment; EVG denotes the empty vector group in which the PC-12 cells were transfected with plasmid vectors without gene fragments; OE means the group in which the PC-12 cells were transfected with human APP plasmid; and BCP denotes the group in which the PC-12 cells were transfected for 48 h and then incubated with different concentrations of BCP for 24 h. **(A)** Effects of different concentrations of BCP on the viability of PC-12 cells for 24 h. **(B)** After 48 h of transfection, the effect of BCP on the viability of PC-12 cells for 24 h. Compared with CN, *^ns^ p* > 0.05. Compared with EVG, *p < 0.05, **p < 0.01. Compared with OE group, ^Δ^*p* < 0.05, ^ΔΔ^*p* < 0.01. One-way ANOVA with Bonferroni’s *post hoc* test.

Cell viability of PC-12 cells incubated with BCP for 24 h was detected using CCK-8. The results indicated no significant difference between the 5 μM BCP group and control group. The groups with 10 and 20 μM of BCP displayed significantly improved cell viability compared with the control group. Compared with the control group, the 25 μM BCP group was found to have significantly enhanced cell viability (Table 2 and Figure 2A), indicating that BCP can increase the viability of PC-12 cells.

**TABLE 2:** Effects of different concentrations of BCP on the 24-h viability of PC-12 cells.

Treatment	Neuronal survival (% CCK-8 reduction)
CN^a^	98.06 ± 0.64
5 μM BCP^b^	100.2 ± 0.65^ns^
10 μM BCP^b^	102.8 ± 0.62*
15 μM BCP^b^	103.3 ± 0.86*
20 μM BCP^b^	104.2 ± 0.76*
25 μM BCP^b^	106.5 ± 0.79**

*^a^The control group without special treatment.*

*^b^The group incubated with different concentrations of BCP for 24 h.*

*ns: no significant difference vs. CN.*

**p < 0.01, * p < 0.05 vs. CN.

Furthermore, our results indicated that BCP can improve the viability of PC-12 cells following transfection. Compared with that in the OE group, the number of cells in the 5, 10, and 20 μM BCP groups increased by 3.1, 14.6, and 17.8%, respectively. The viability of PC-12 cells overexpressing APP gene was significantly reduced when compared with that of the vector group. Compared with the OE group, the 10 and 20 μM BCP groups displayed substantially improved cell viability (Table 3 and Figure 2B).

**TABLE 3:** After 48 h of transfection, the effect of BCP on the viability of PC-12 cells for 24 h.

Treatment	Neuronal survival (% CCK-8 reduction)
CN^a^	100.0 ± 1.09
EVG^b^	91.25 ± 5.6^ns^
OE^c^	30.23 ± 1.8**
5 μM BCP^d^	31.18 ± 1.6
10 μM BCP^d^	34.66 ± 0.3^Δ^
20 μM BCP^d^	35.61 ± 0.5^Δ^

*^a^The control group without special treatment.*

*^b^The empty vector group, in which the PC-12 cells were transfected with plasmid vectors without gene fragments.*

*^c^The group in which the PC-12 cells were transfected with human APP plasmid.*

*^d^The group in which the PC-12 cells were transfected for 48 h and then incubated with different concentrations of BCP for 24 h.*

*ns: no significant difference vs. CN.*

**p < 0.01 vs. EVG.

*^Δ^p < 0.05 vs. OE.*

### β-Caryophyllene Improved the Neurite Morphology of Amyloid-β Protein Precursor Overexpressed PC-12 Cells

**FIGURE 3:**
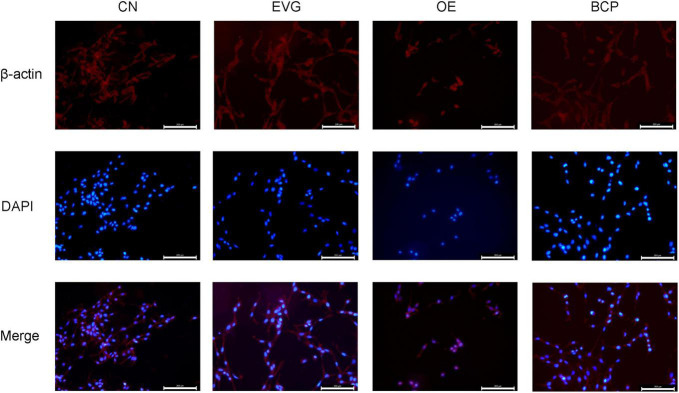
Immunofluorescence detection of the effect of β-caryophyllene on the neurites of PC-12 cells with APP overexpression. CN denotes the control group without special treatment; EVG denotes the group in which the PC-12 cells were transfected with empty vectors plasmid for 72 h; and OE denotes the group in which the PC-12 cells were transfected with human APP plasmid for 72 h. In the BCP group, the PC-12 cells were transfected for 48 h to overexpress APP and 10 μM BCP acted on the PC-12 cells for 24 h. Scale bar = 200 μm.

By using immunofluorescence to examine the synaptic morphology of PC-12 cells, researchers have discovered the phenomena of cell rounding and synaptic loss after 72 h of PC-12 cell transfection with a plasmid containing APP cDNA. As shown in Figure 3, when the EVG group was compared with the OE group, the OE group cells were found to be significantly rounded, with the neurite being lost; however, the BCP group’s neurite morphology was found to be improved.

### β-Caryophyllene Inhibited the Expressions of JAK2, Amyloid-β Protein Precursor, STAT3, and BACE1 in Amyloid-β Protein Precursor Overexpressed PC-12 Cells

**FIGURE 4:**
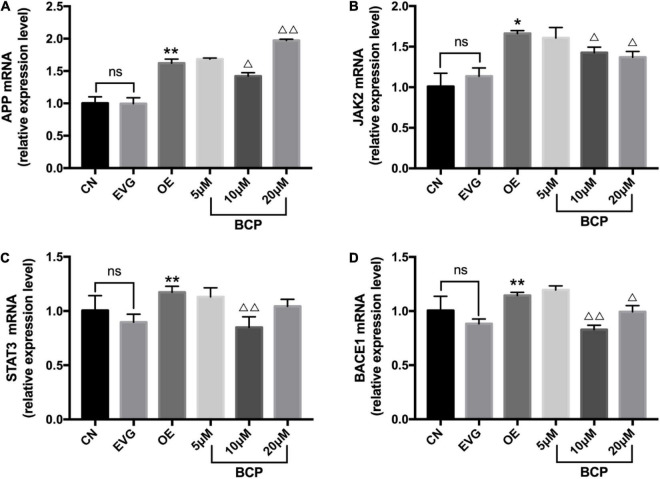
Expressions of JAK2, APP, STAT3, and BACE1 mRNA in the PC-12 cells. CN denotes the control group without special treatment; EVG denotes the empty vector group in which the PC-12 cells were transfected with plasmid vectors without gene fragments; OE denotes the group in which the PC-12 cells were transfected with a plasmid containing APP cDNA; and BCP denotes the group in which the effect of BCP was assessed on 24-h viability of PC-12 cells transfected for 48 h. **(A)** Relative expression level of APP mRNA. **(B)** Relative expression level of JAK2 mRNA. **(C)** Relative expression level of STAT3 mRNA. **(D)** Relative expression level of BACE1 mRNA. Compared with CN, *^ns^ p* > 0.05. Compared with EVG, *p < 0.05, **p < 0.01. Compared with OE group, ^Δ^*p* < 0.05, ^ΔΔ^*p* < 0.01. One-way ANOVA with Bonferroni’s *post hoc* test.

After applying BCP (5, 10, and 20 μM) to the nerve injury model for 24 h, the expression levels of JAK2, APP, STAT3, and BACE1 mRNA were determined using the qRT-PCR assay; the results are depicted in Figure 4. APP, JAK2, BACE1, and STAT3’s mRNA levels in the OE group increased significantly compared with those in the EVG group.

Compared with the OE group, the 5 μM BCP group exhibited no significant difference, the 10 μM BCP group exhibited significantly downregulated APP, JAK2, BACE1, and STAT3 expressions at the mRNA level, and the 20 μM BCP group exhibited significantly downregulated JAK2 and BACE1 expressions at the mRNA levels (Figure 4).

**FIGURE 5:**
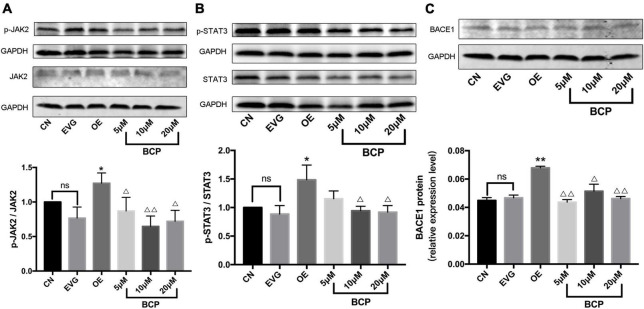
Effect of β-caryophyllene on JAK2 and STAT3 phosphorylation and BACE1 protein expression in PC-12 cells. CN denotes the control group without special treatment; EVG denotes the empty vector group in which the PC-12 cells were transfected with plasmid vectors without gene fragments; OE denotes the group in which the PC-12 cells were transfected with a plasmid containing APP cDNA; and BCP denotes the group in which the effect of BCP on the 24-h viability of PC-12 cells transfected for 48 h was assessed. **(A)** Phosphorylation level of JAK2 protein. **(B)** Phosphorylation level of STAT3 protein. **(C)** Relative expression level of BACE1 protein. Compared with CN, *^ns^ p* > 0.05. Compared with EVG, *p < 0.05, **p < 0.01. Compared with OE group, ^Δ^*p* < 0.05, ^ΔΔ^*p* < 0.01. One-way ANOVA with Bonferroni’s *post hoc* test.

The phosphorylation level of JAK2 protein in the OE group was significantly higher than that in the EVG group. The phosphorylation level of JAK2 protein was significantly lower in the 5, 10, and 20 μM BCP groups than in the OE group. Figure 5A depicts the aforementioned results.

The phosphorylation level of STAT3 protein in the OE group was significantly higher than that in the EVG group. The phosphorylation level of STAT3 protein in the BCP (5, 10, and 20 μM) groups was lower than that in the OE group, whereas the phosphorylation level of STAT3 protein in the 10 and 20 μM BCP groups was more significant (Figure 5B).

The expression level of BACE1 protein in the OE group was upregulated compared with that in the EVG group. The 5 and 20 μM BCP groups displayed a considerably downregulated BACE1 protein expression level, whereas the 10 μM BCP group displayed significantly downregulated BACE1 protein expression (Figure 5C).

### Molecular Docking Simulation of β-Caryophyllene With JAK2, STAT3, BACE1, and Cannabinoid Receptor Type 2 Proteins

The estimated ΔG values of the docking model were obtained through molecular docking, and the estimated ΔG values of JAK2, STAT3, and BACE1 were compared with those of the positive protein CB2 of BCP to predict if the protein interacts with BCP. The larger the negative value, the more stable is the docking model. Table 4 displays the docking data, indicating that (+)-BCP has the best docking stability with JAK2.

**TABLE 4:** Results of molecular docking.

*NO.*	Target	uniprot entrez id	pdb id	Ligand	Estimated ΔG kcal/mol
1	JAK2	O60674	5L3A	(+)-β-Caryophyllene	−6.87
2	CB2^a^	P34972	6KPC	(+)-β-Caryophyllene	−6.75
3	CB2^a^	P34972	6KPC	(−)-β-Caryophyllene	−6.74
4	STAT3	P40763	5AX3	(−)-β-Caryophyllene	−6.62
5	JAK2	O60674	5L3A	(−)-β-Caryophyllene	−6.60
6	STAT3	P40763	5AX3	(+)-β-Caryophyllene	−6.49
7	BACE1	P56817	7DCZ	(+)-β-Caryophyllene	−6.49
8	BACE1	P56817	7DCZ	(−)-β-Caryophyllene	−6.45

*^a^cannabinoid receptor type 2.*

**FIGURE 6:**
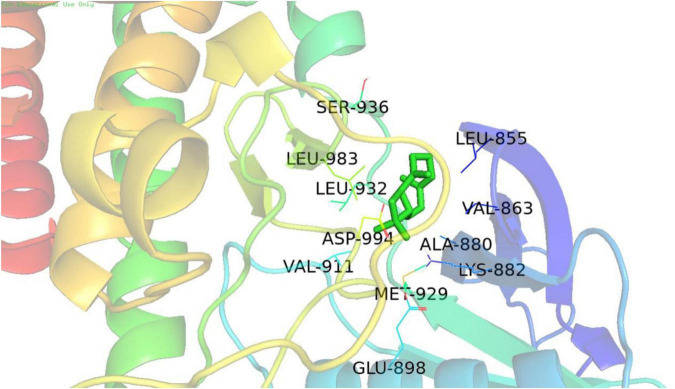
The best docking stability model. Model of the putative interaction of (+)-β-caryophyllene with the JAK2 receptor determined.

Molecular docking analysis revealed that (−)-BCP binds to the JAK2 water accessible cavity’s hydrophobic region (Figure 6). In this model, (−)-BCP was docked into the hydrophobic binding cavity of the docking pocket calculated by Autodock Vina to form alkyl hydrophobic interactions with the amino acid residues val-911, lys-882, ala-880, met-929, leu-983, val-863, and leu-855, as well as van der Waals interactions with the amino acid residues asp-994, gly-993, leu-332, gly-935, and gly-856.

## Discussion

Alzheimer’s disease has been causing a substantial health burden, and its prevalence is increasing with global population ageing (Urfer et al., 2021). After decades of research, scientists have concluded that Aβ extracellular plaques are directly linked to the neuropathological events such as synaptic and neuronal cell death (Cisternas et al., 2020; Fracassi et al., 2020). According to research, increasing Aβ oligomer formation triggers neuronal dysfunction and network alternations in learning and memory circuitry prior to the clinical onset of AD, and inhibition of Aβ accumulation improves cognitive behaviour and neuroinflammation in AD mice (Jiang et al., 2021). We utilised PC-12 cells overexpressing the APP gene to construct an *in vitro* AD model. At 72 h after transfection, the cell morphology altered significantly, becoming rounder, and the number of cells decreased. Moreover, we found that the relative expression levels of JAK2, STAT3, and BACE1 mRNA were considerably upregulated in the transfected PC-12 cells of human APP cDNA.

Activation of the “p-JAK2-p-STAT3-NF-κB-BACE1” pathway has been linked to Aβ accumulation and neurotoxicity (Park et al., 2016). The “JAK2-STAT3” pathway can mediate the expression of endogenous BACE1. BACE1 is an APP cleavage protein that is required for APP processing to Aβ (Ye et al., 2017) and is a promising therapeutic target for reducing Aβ production in early AD. BACE1 has other substrates outside the amyloidogenic pathway that may be essential for synaptic plasticity and synaptic homeostasis (Hampel et al., 2021), which has been one of the most crucial research topics for scientists.

Our team investigated the neuroprotective effects of BCP and discovered that it may considerably improve PC-12 cell viability while also protecting the cell morphology. Further investigation showed that BCP can inhibit APP, JAK2, STAT3, BACE1 mRNA and protein overexpression, as well as JAK2 and STAT3 hyperphosphorylation. We discovered that 10 and 20 μM BCP may lower BACE1 protein expression, implying that BCP has great potential in AD treatment.

β-Caryophyllene has been shown to inhibit neuronal death. In a study by Yujie Cheng, the oral administration of BCP prevented cognitive impairment in APP/PS1 mice, and this positive cognitive effect was associated with reduced β-amyloid burden in both hippocampus and cerebral cortex (Cheng et al., 2014). The Rui Wang team discovered that miR-433, which targets JAK2, was downregulated in both AD serum and SH-SY5Y cells treated with Aβ (Wang and Zhang, 2020). In addition, a study reported that the “JAK2-STAT3” pathway activation inhibits NSC neurogenesis, whereas inhibition of the “JAK2-STAT3” pathway improves memory deficit in AD mice (Kong et al., 2019). To investigate whether the neuroprotective effect of BCP is related to the “JAK2-STAT3-BACE1” pathway, we utilised molecular docking simulation, a well-established *in silico* structure-based approach, which is extensively used in drug discovery (Pinzi and Rastelli, 2019). The molecular docking field has been advancing, with new algorithms and methods appearing at an exponential rate, making it helpful in accurately determining the mechanism of ligand-protein interaction. Because BCP exhibits selective full agonism on cannabinoid receptor type 2 (CB2) (Hashiesh et al., 2020), we chose CB2 as a positive protein for molecular docking and discovered that BCP and JAK2 have good docking stability. Prediction of the interaction between biological targets and ligands through molecular docking provides ideas for the follow-up study on the mechanism of BCP protecting nerve cells, which can provide more possibilities for AD treatment.

The results suggested that BCP might reduce BACE1 expression by inhibiting JAK2 phosphorylation, which is consistent with western blot results. Recent studies suggest that BCP has anti-inflammatory and antioxidant effects that apromote neuroprotection in different cognitive damage animal models (Chávez-Hurtado et al., 2020). In addition, we found that BCP exerts a positive effect on reducing Aβ load, which indicates its significance in the treatment of AD. To explore the function mechanism of BCP antagonizing Aβ, it is necessary to further study and clarify the relationship between BCP and receptors of the “JAK2-STAT3-BACE1” signalling pathway.

## Conclusion

Modern research mostly focuses on the action mechanism of BCP in improving neuroinflammation and cognitive decline (Lindsey et al., 2019). This study investigated the notable effect of BCP on protecting nerve cells and APP processing to Aβ. The essential protein BACE1 and the phosphorylation level of the “JAK2-STAT3” pathway decreased significantly when BCP was incubated with the cells transfected with human APP plasmid. Therefore, exploring the potential of BCP in reducing the damage to nerve cells caused by Aβ holds great significance. Based on the outcome of various experiments, we concluded that “JAK2-STAT3-BACE1” pathway inhibition might be one of the avenues for investigating BCP’s neuroprotective effects and antagonism of Aβ’s neurotoxicity.

## Data Availability Statement

The original contributions presented in the study are included in the article/Supplementary Material, further inquiries can be directed to the corresponding author/s.

## Author Contributions

YZ conducted experiments and wrote manuscript. SW, HJ, and SH analyzed the data. CL and LJ designed the study. QS and QW guided the experiment. Other authors helped revise the text to the final form. The final manuscript has been read and approved by all authors.

## Conflict of Interest

The authors declare that the research was conducted in the absence of any commercial or financial relationships that could be construed as a potential conflict of interest.

## Publisher’s Note

All claims expressed in this article are solely those of the authors and do not necessarily represent those of their affiliated organizations, or those of the publisher, the editors and the reviewers. Any product that may be evaluated in this article, or claim that may be made by its manufacturer, is not guaranteed or endorsed by the publisher.

## Footnotes

1
www.pymol.org
