# Supplementary figures and images for: The Food Additive β-Caryophyllene Exerts Its Neuroprotective Effects Through the JAK2-STAT3-BACE1 Pathway
Source: Front Aging Neurosci. 2022 Feb 28;14:814432. doi: 10.3389/fnagi.2022.814432 (PMC8919047; doi:10.3389/fnagi.2022.814432)

CN

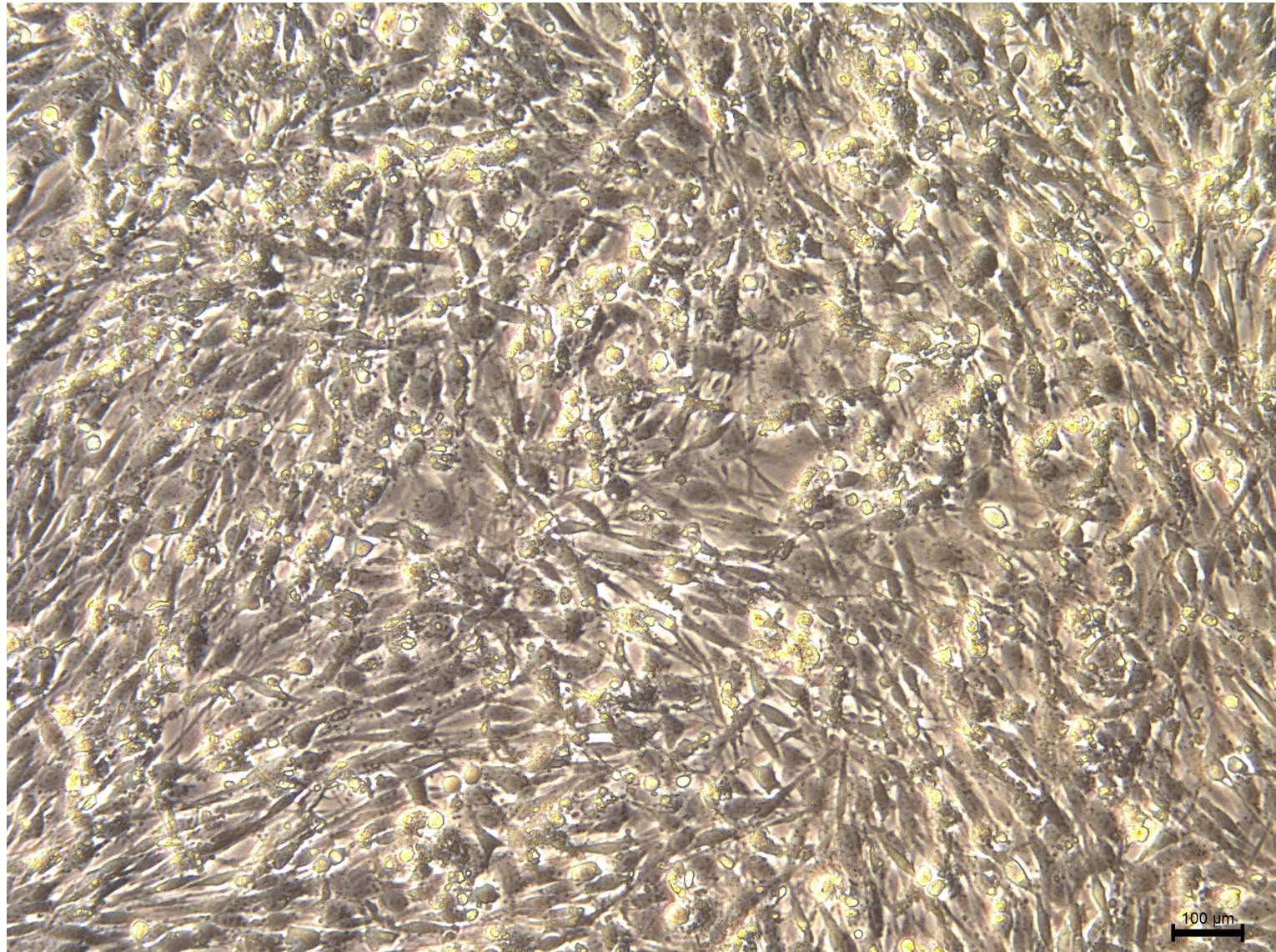

EVG

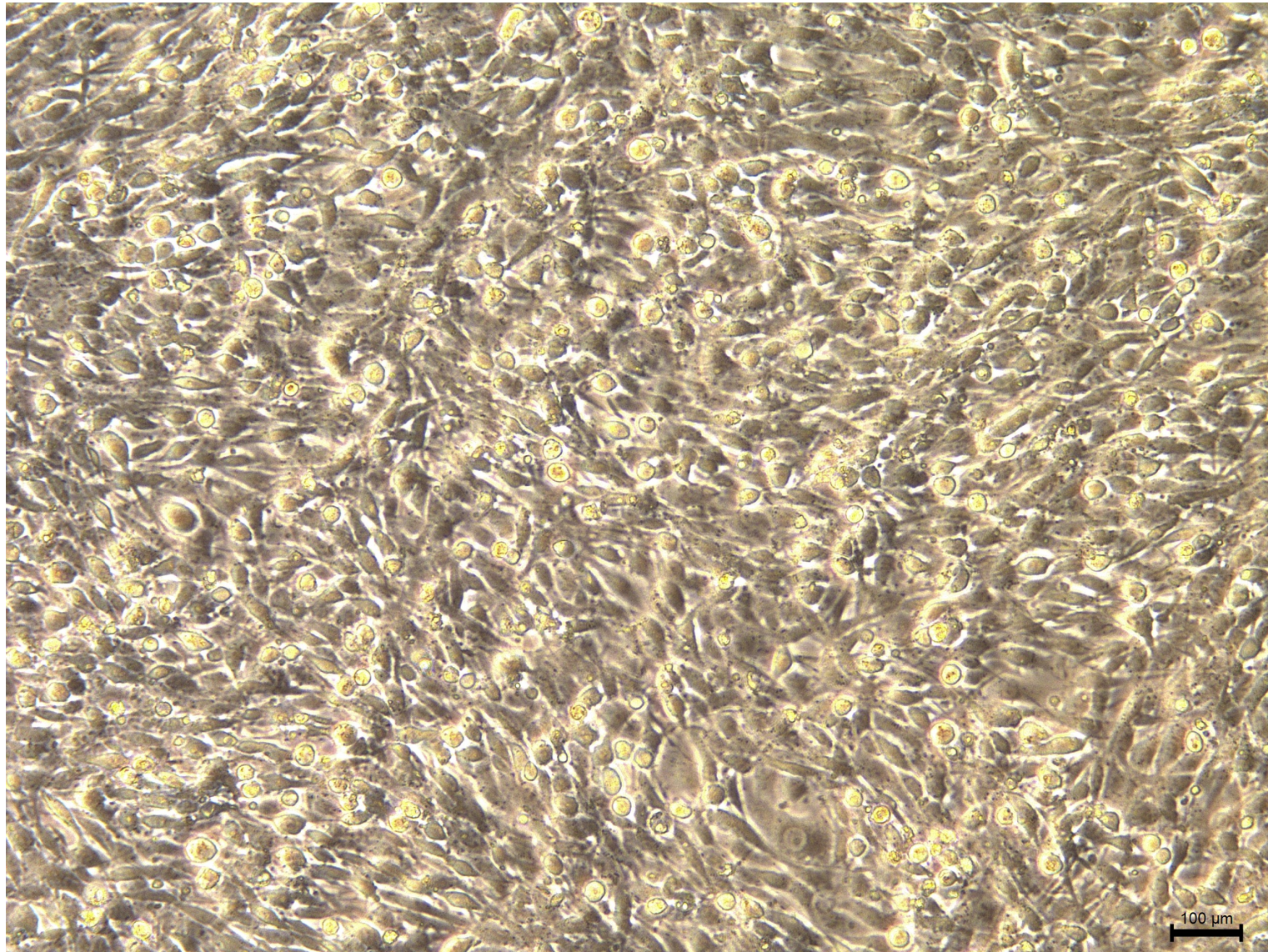

EVG

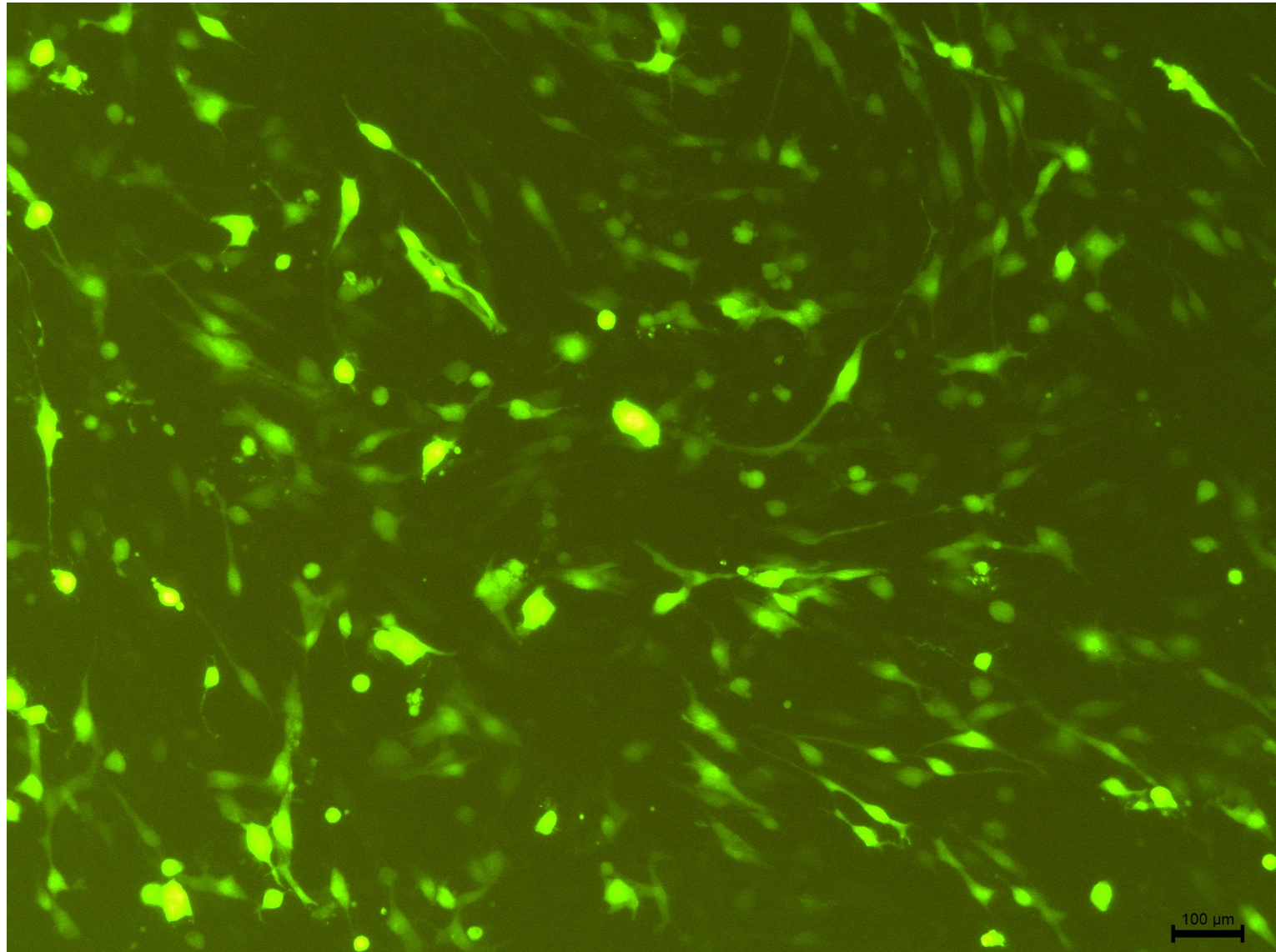

OE

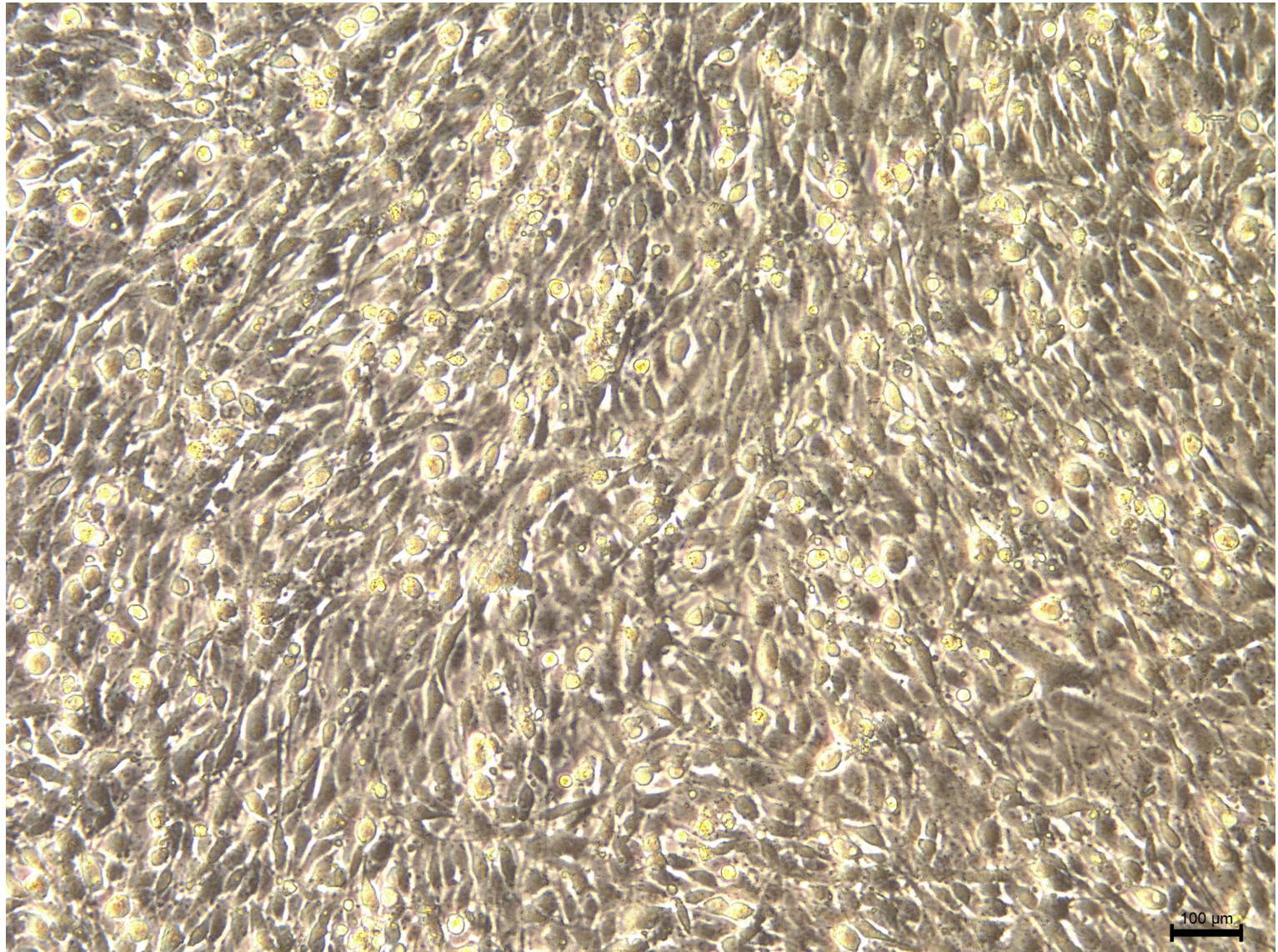

OE

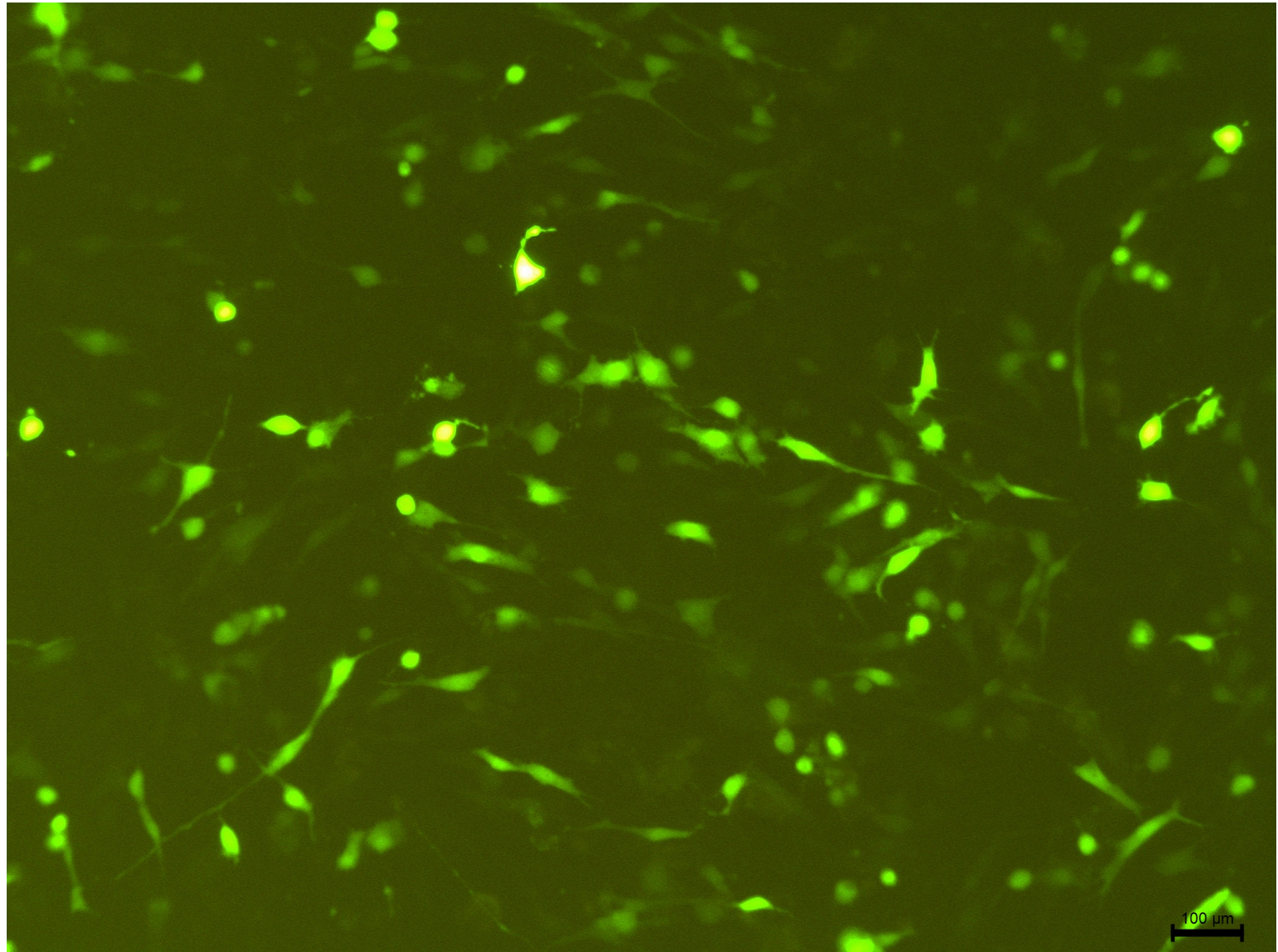

Supplement: Supplementary file 1 [file Data_Sheet_1.PDF]

# Western Blot Initial Data

Figure 1-B

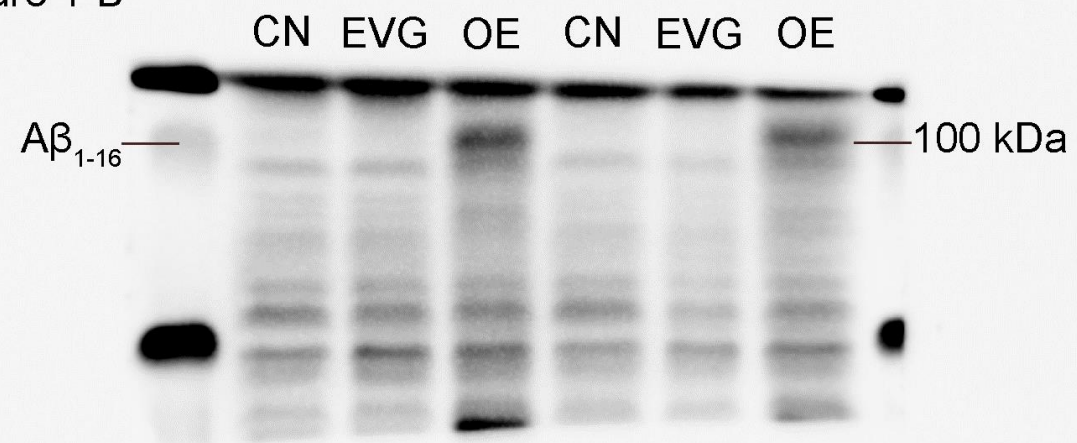

Figure-1B

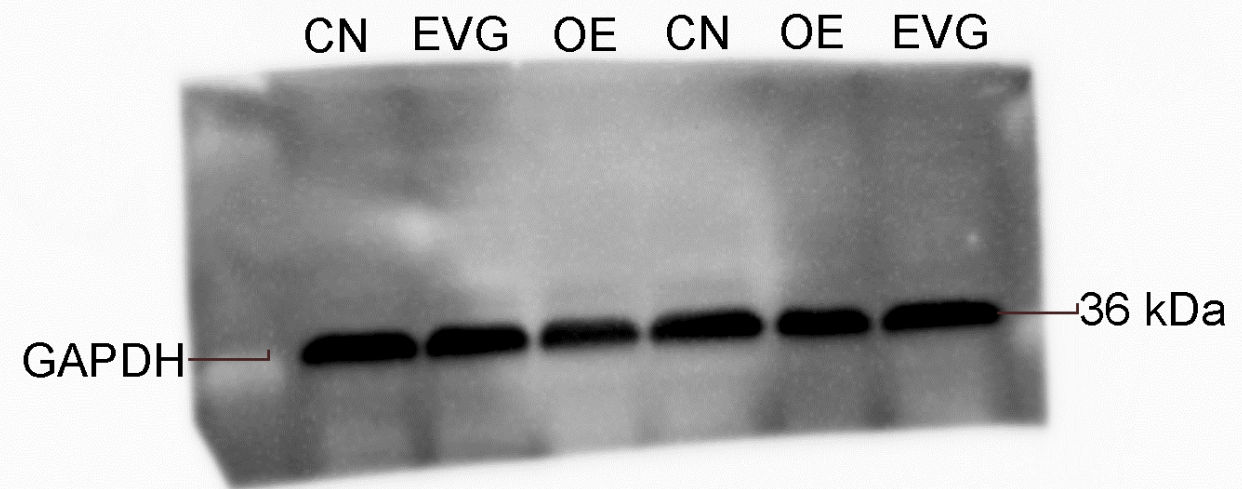

Figure-5A

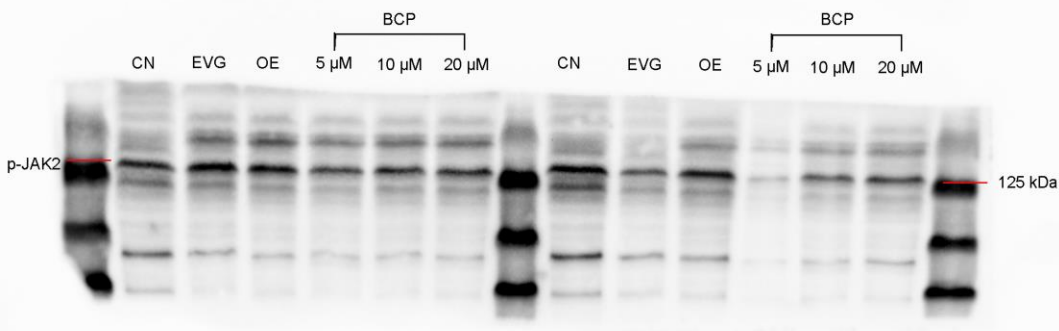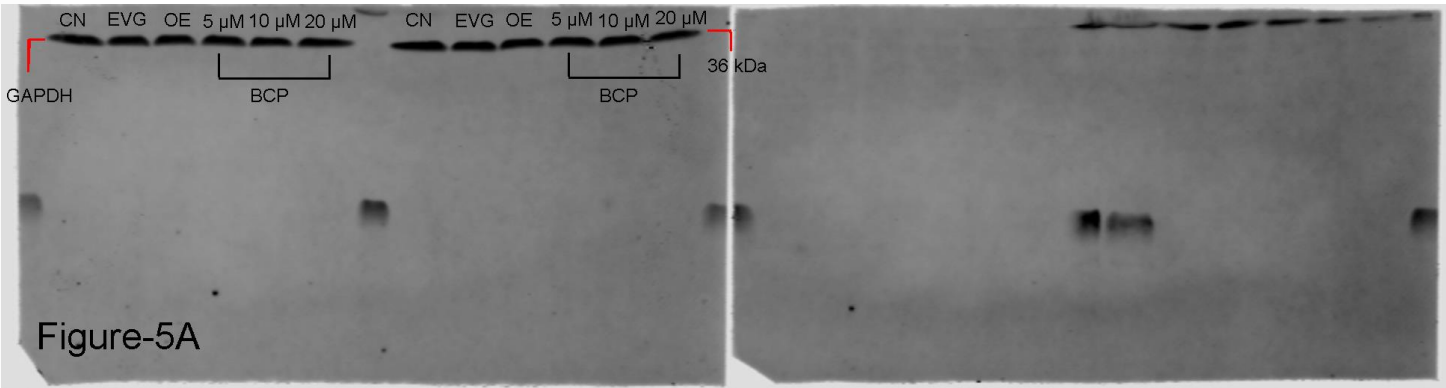

Figure-5A

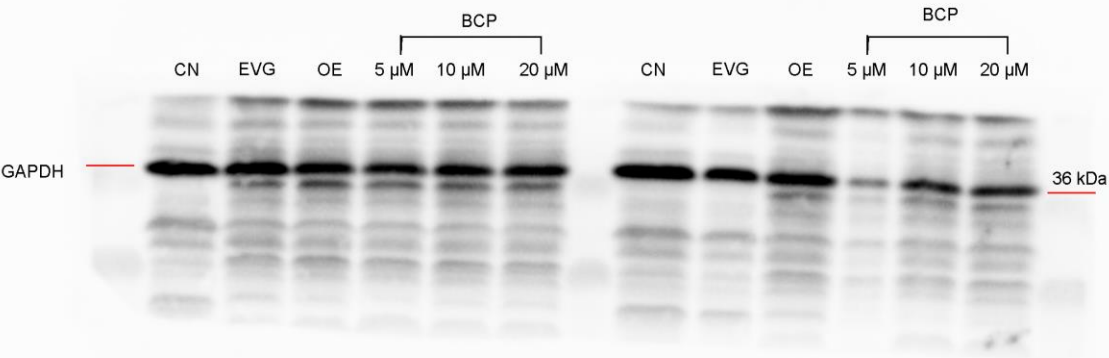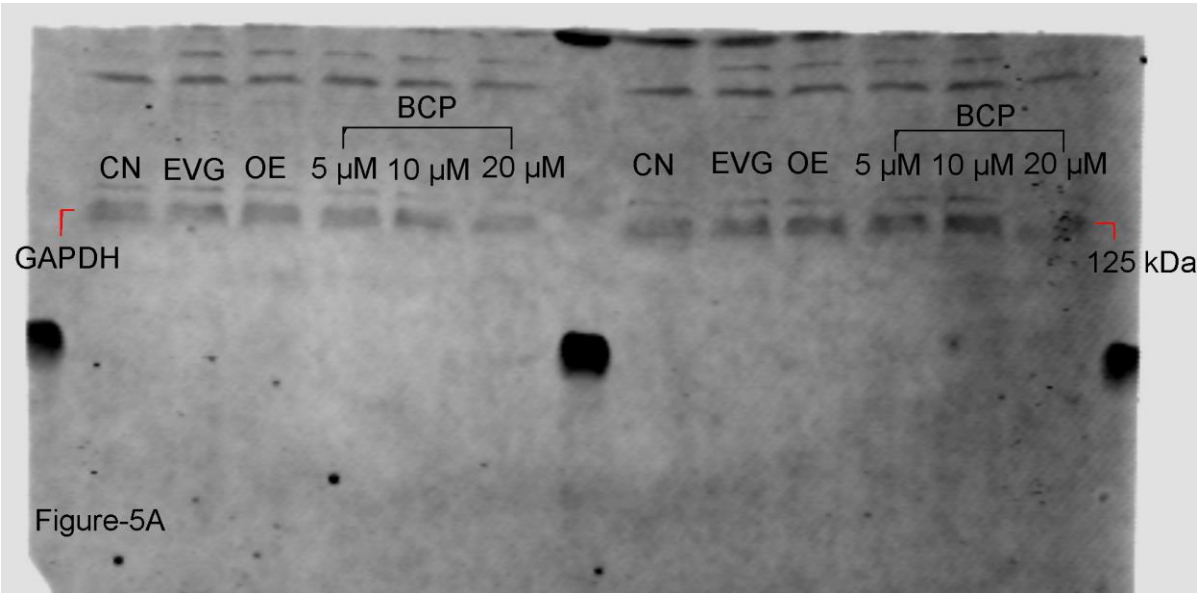

Figure-5B

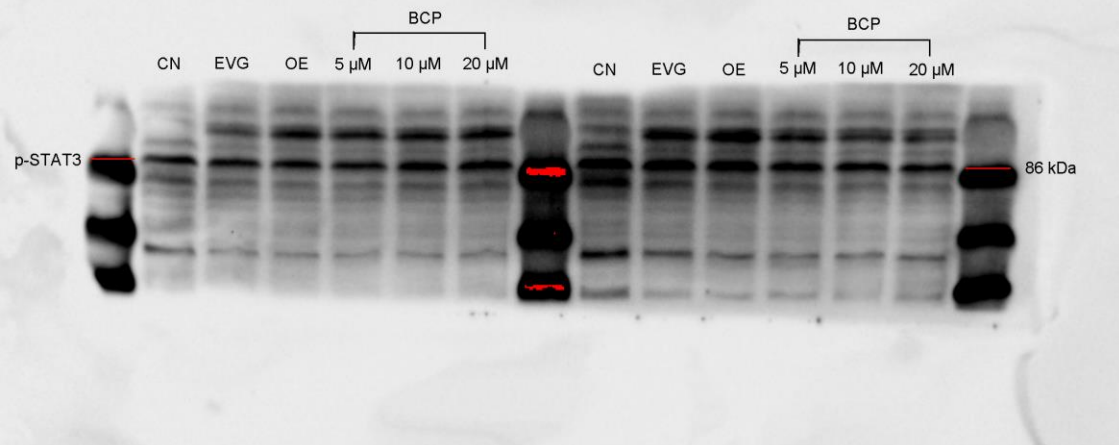

Figure-5B

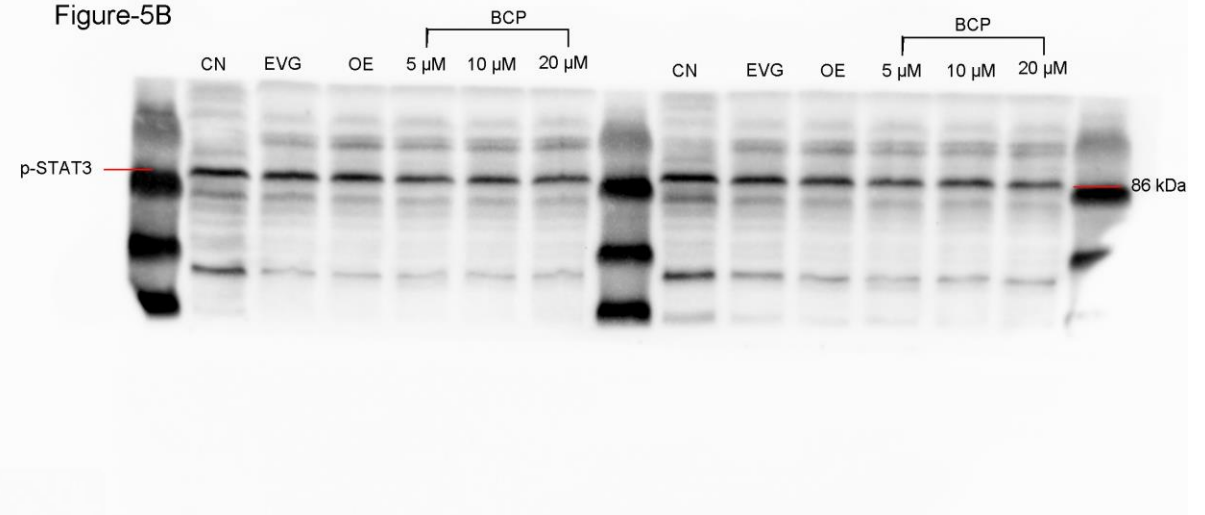

Figure-5B

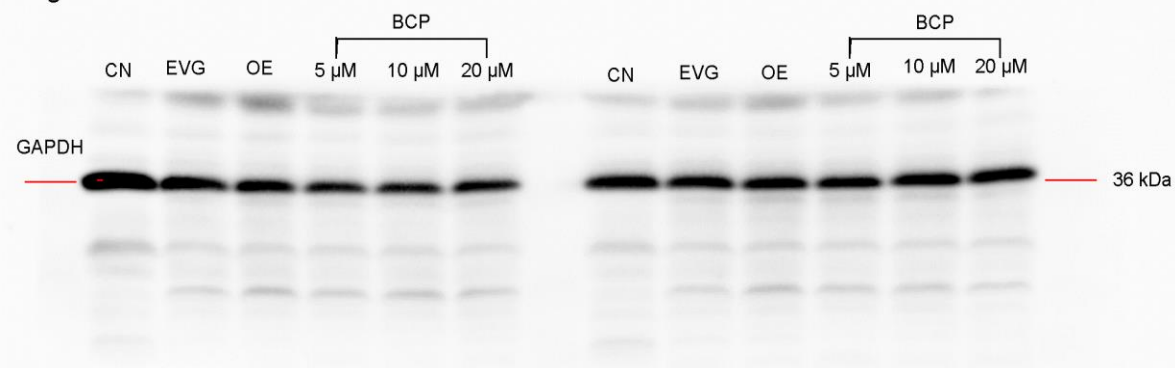

Figure-5B

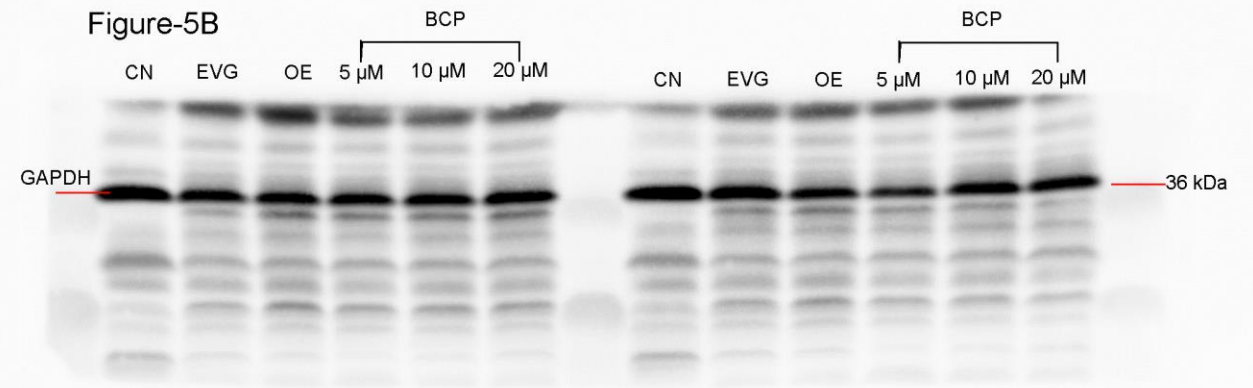

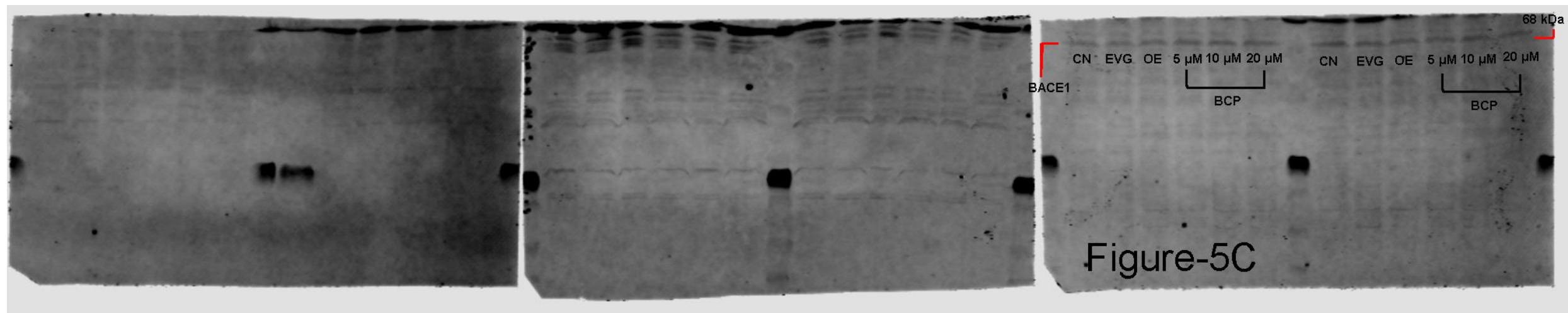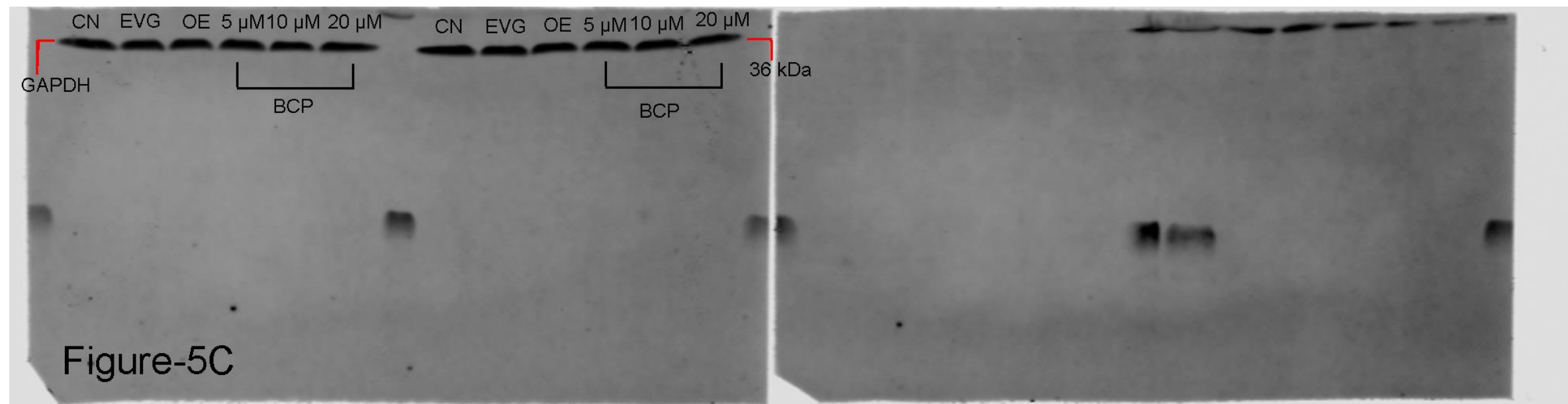

Supplement: Supplementary file 3 [file Data_Sheet_3.PDF]
