# Supplementary material for: The Food Additive β-Caryophyllene Exerts Its Neuroprotective Effects Through the JAK2-STAT3-BACE1 Pathway
Source: Front Aging Neurosci. 2022 Feb 28;14:814432. doi: 10.3389/fnagi.2022.814432 (PMC8919047; doi:10.3389/fnagi.2022.814432)

BC-beta-actin

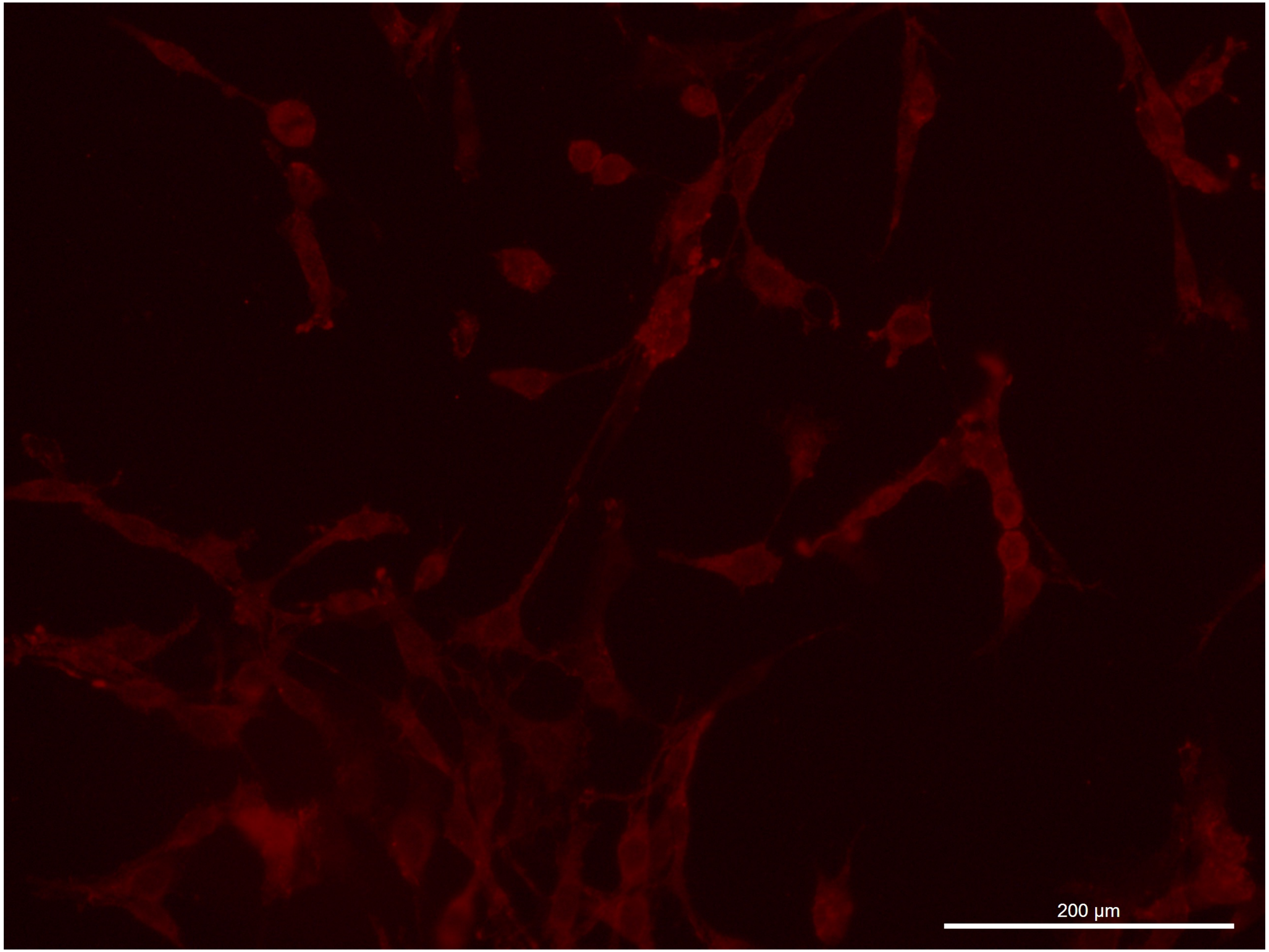

BC-DAPI

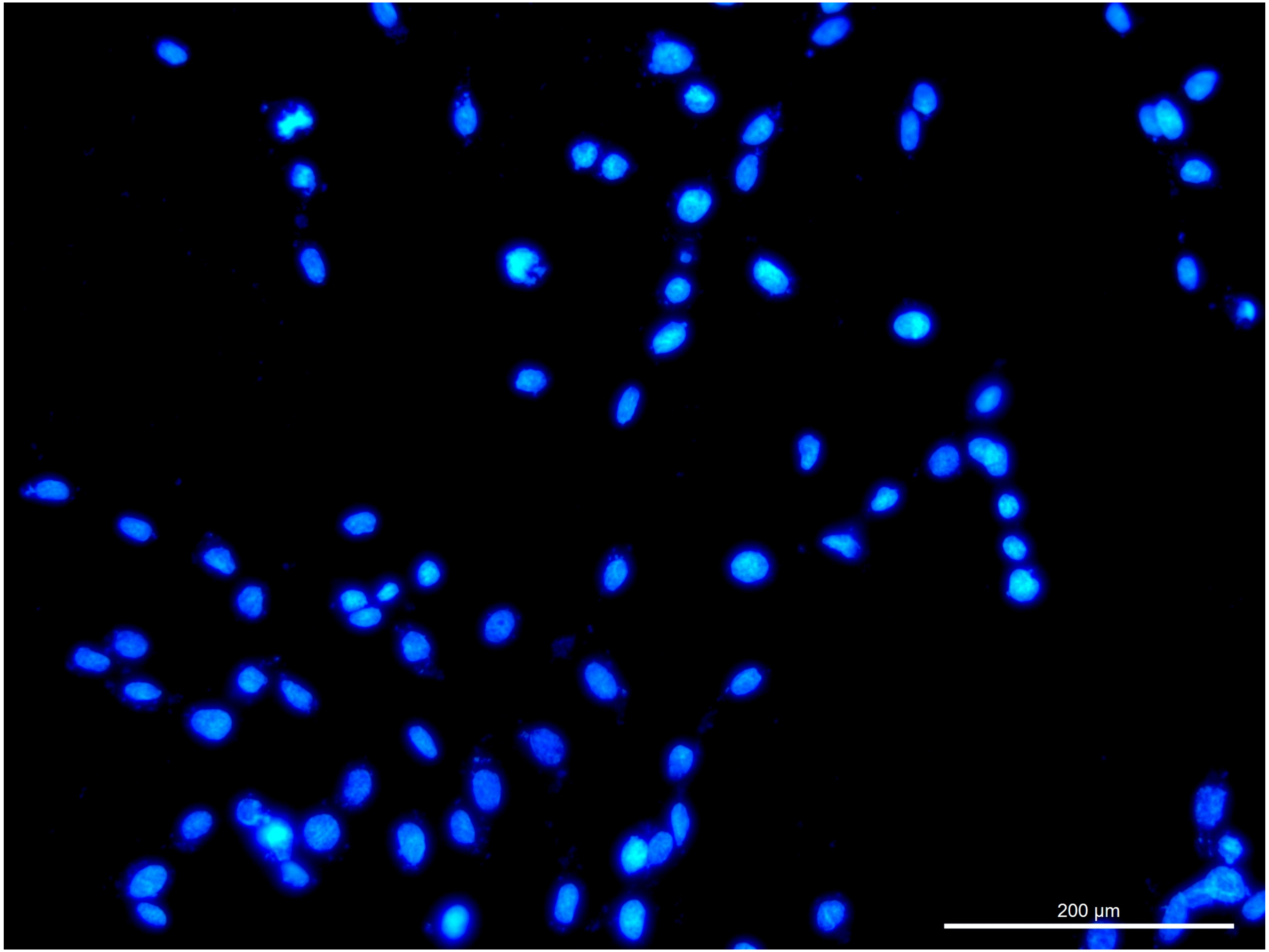

BC-merge

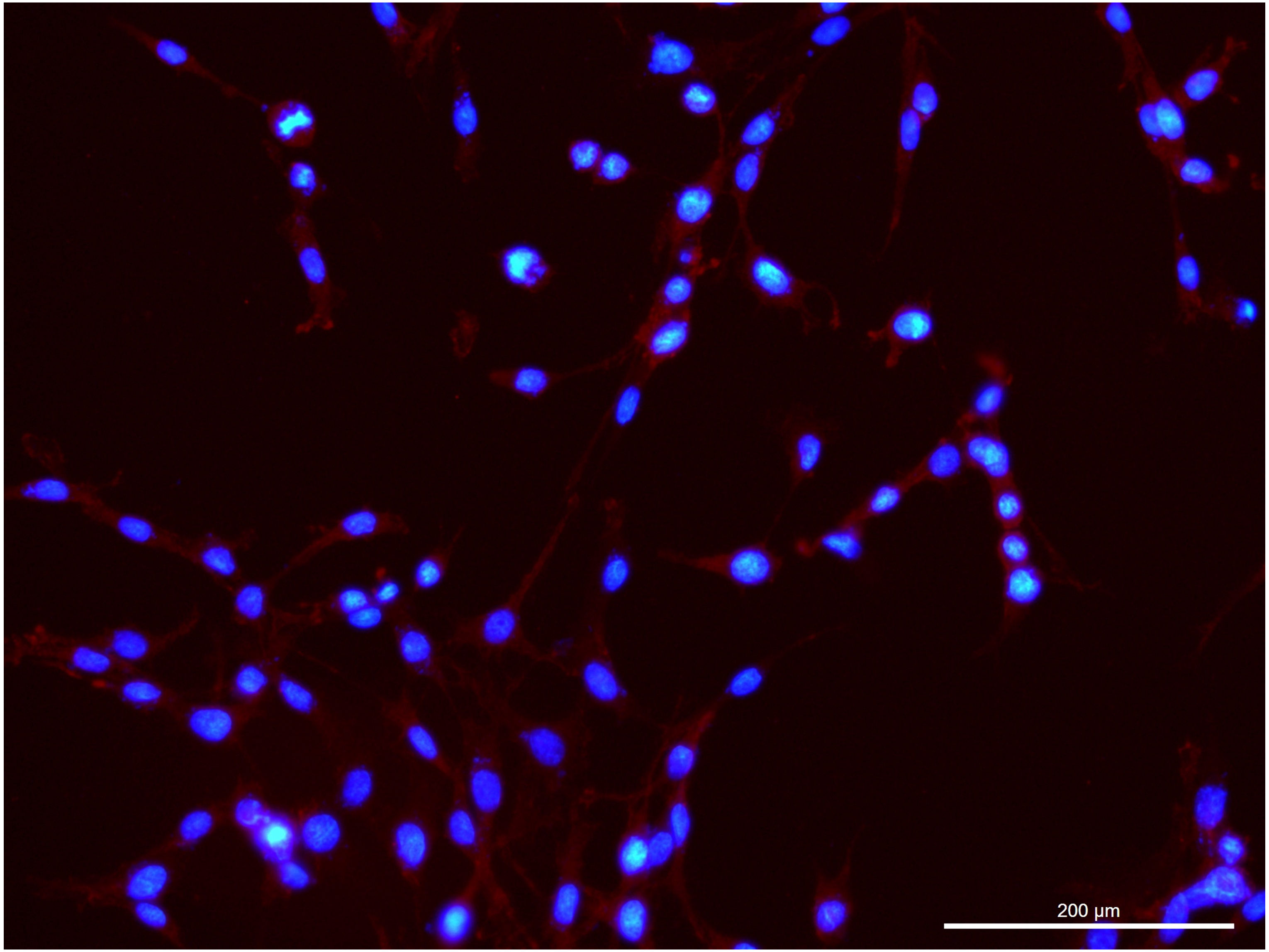

CN-beta-actin

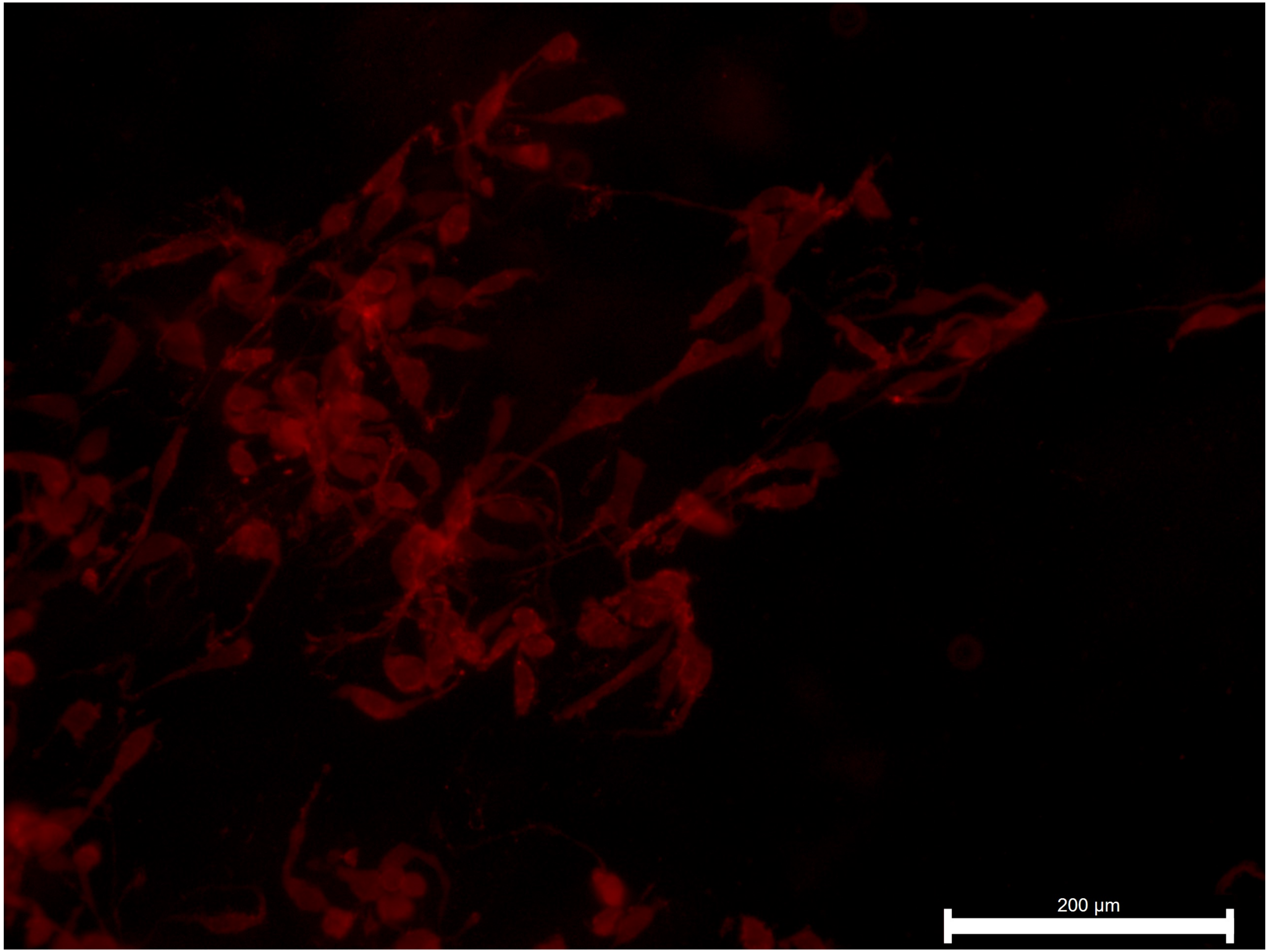

CN-DAPI

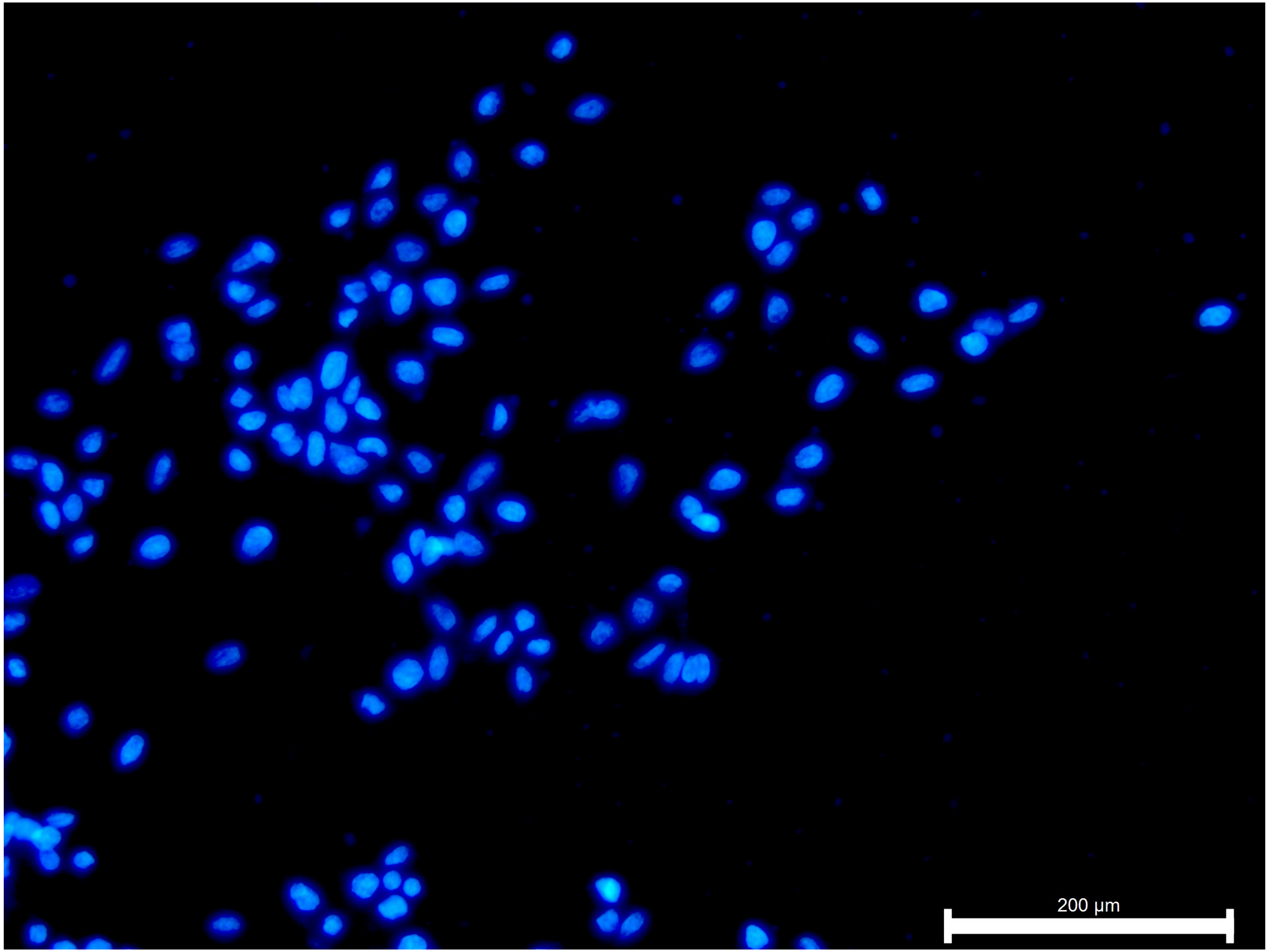

CN-merge

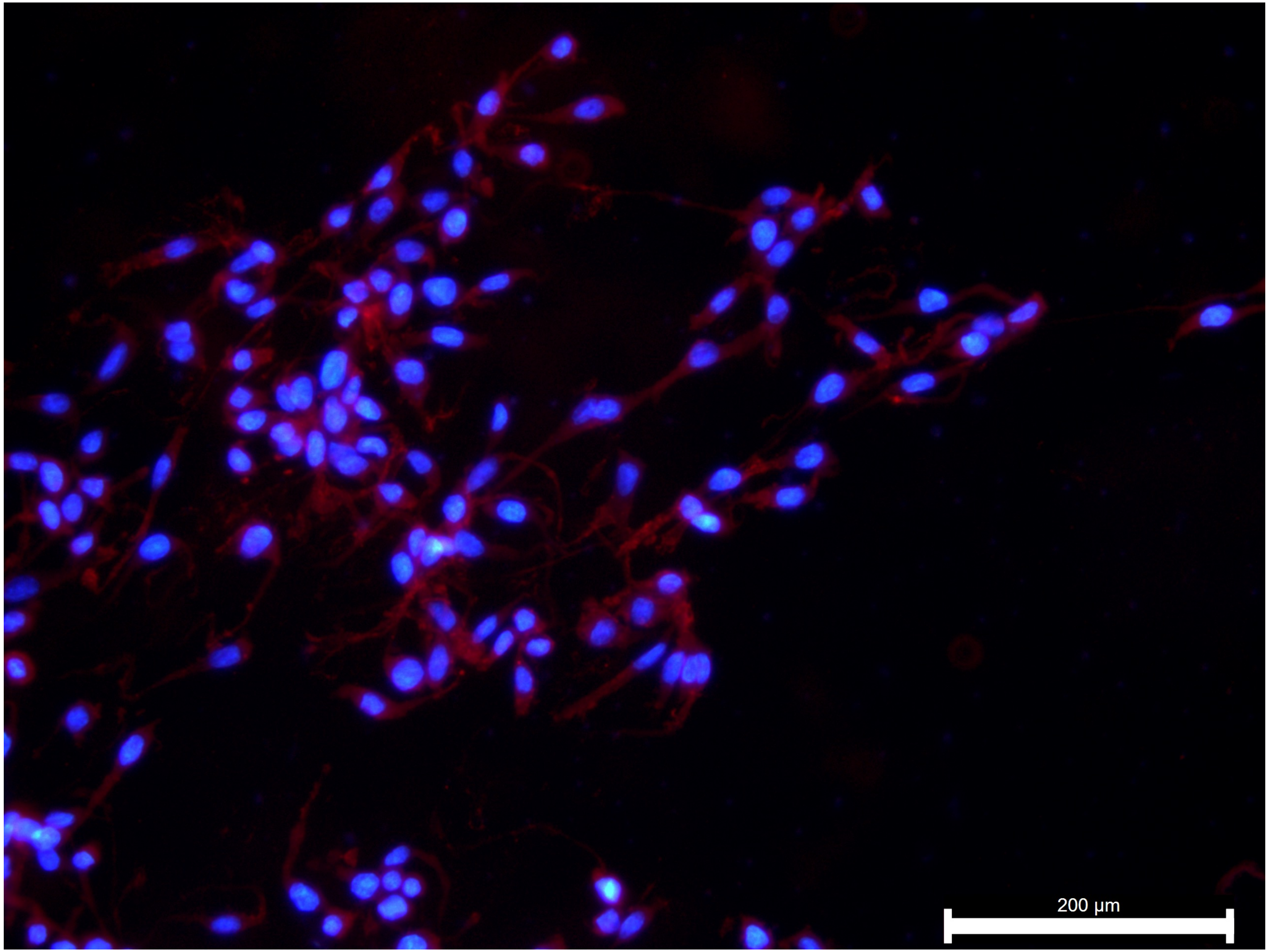

EVG-beta-actin

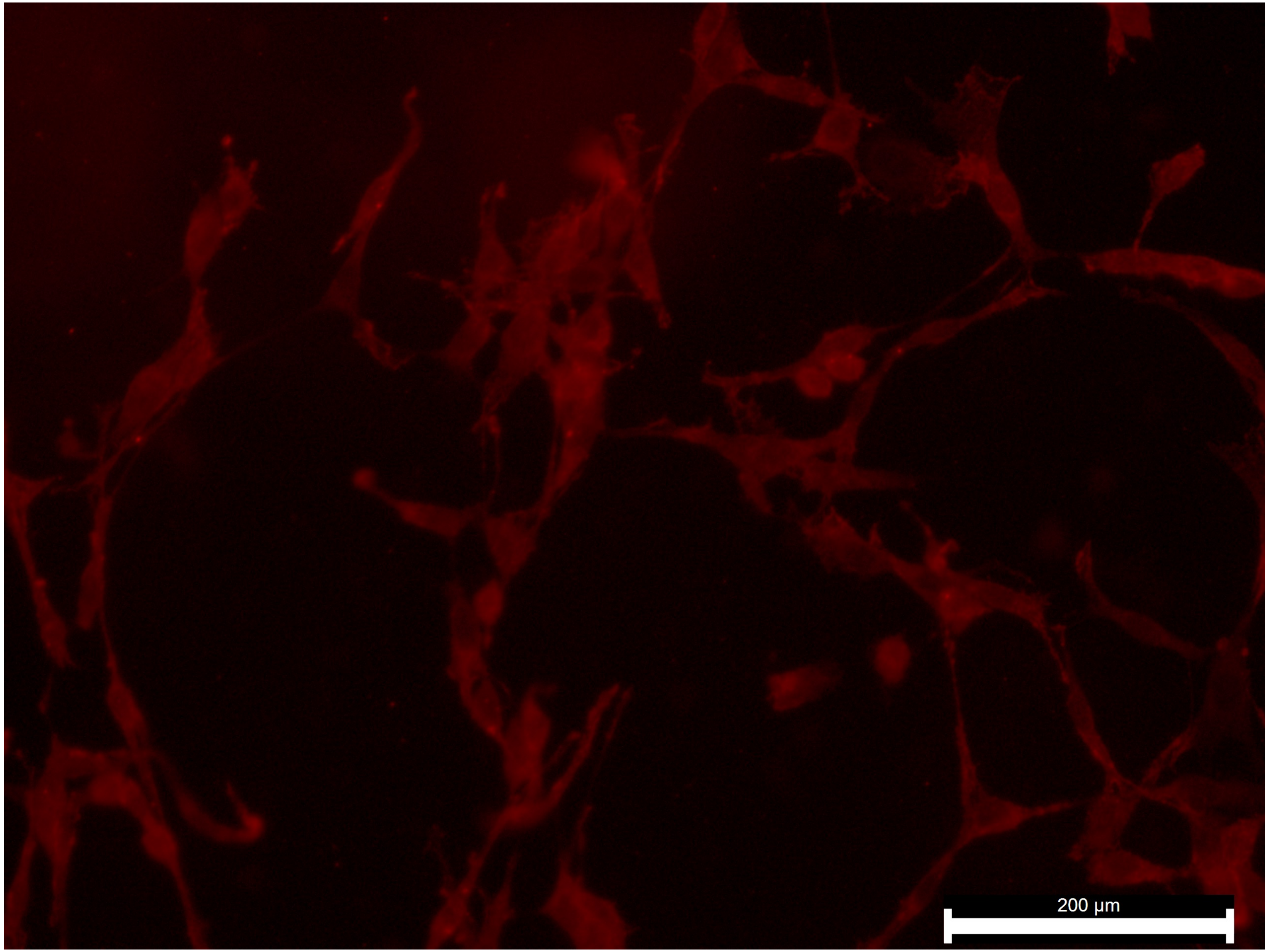

EVG-DAPI

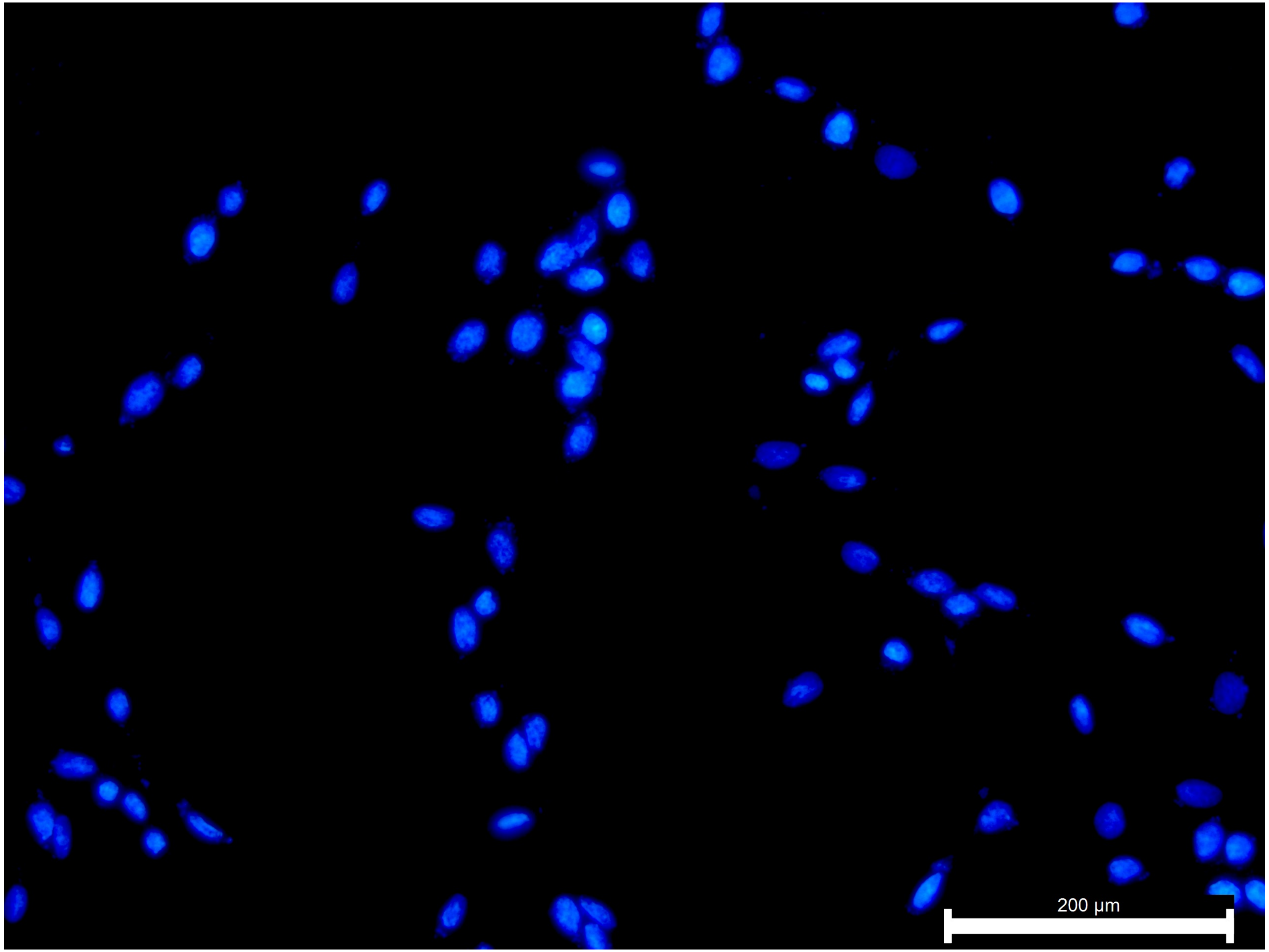

EVG-merge

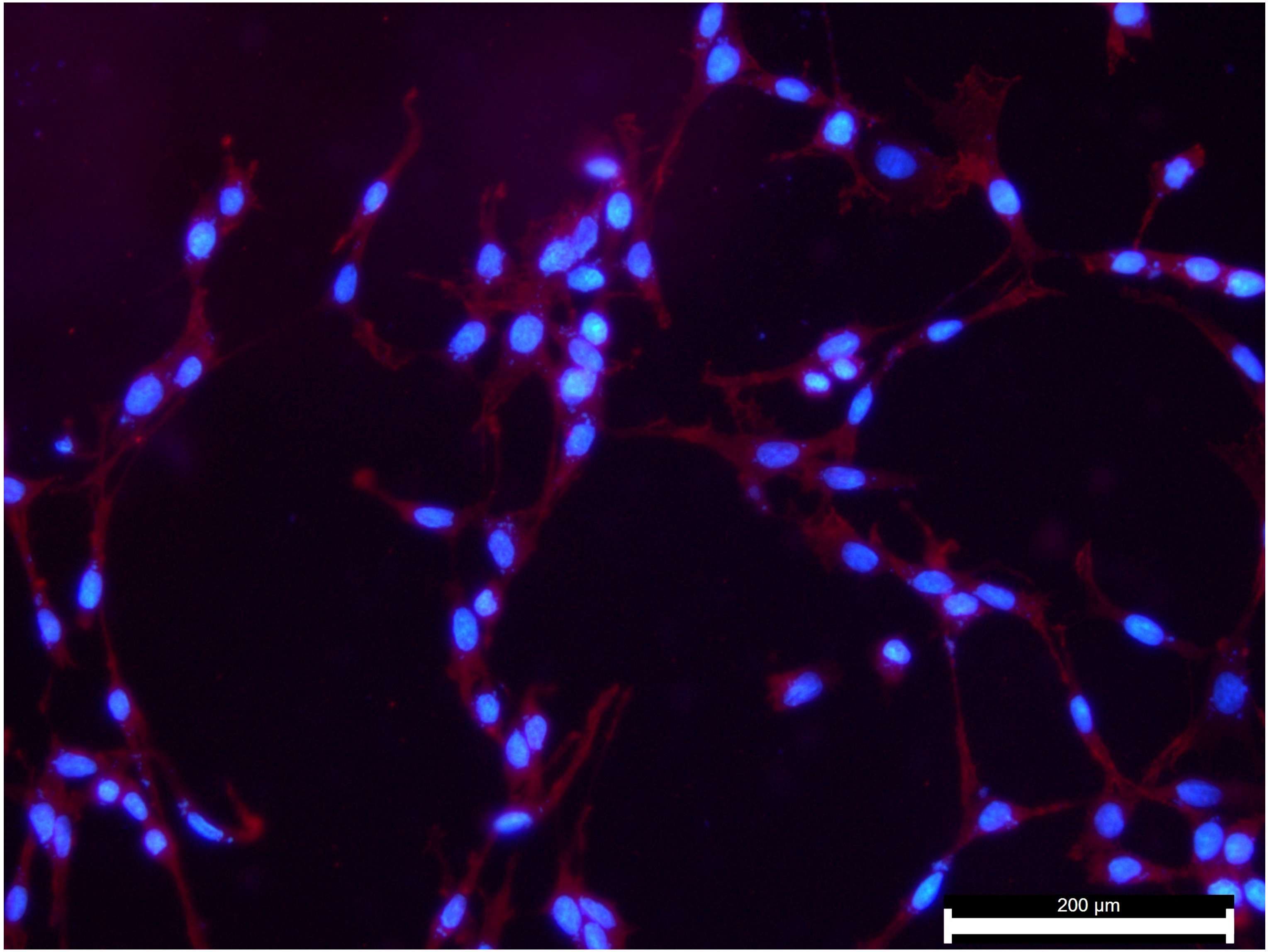

OE-beta-actin

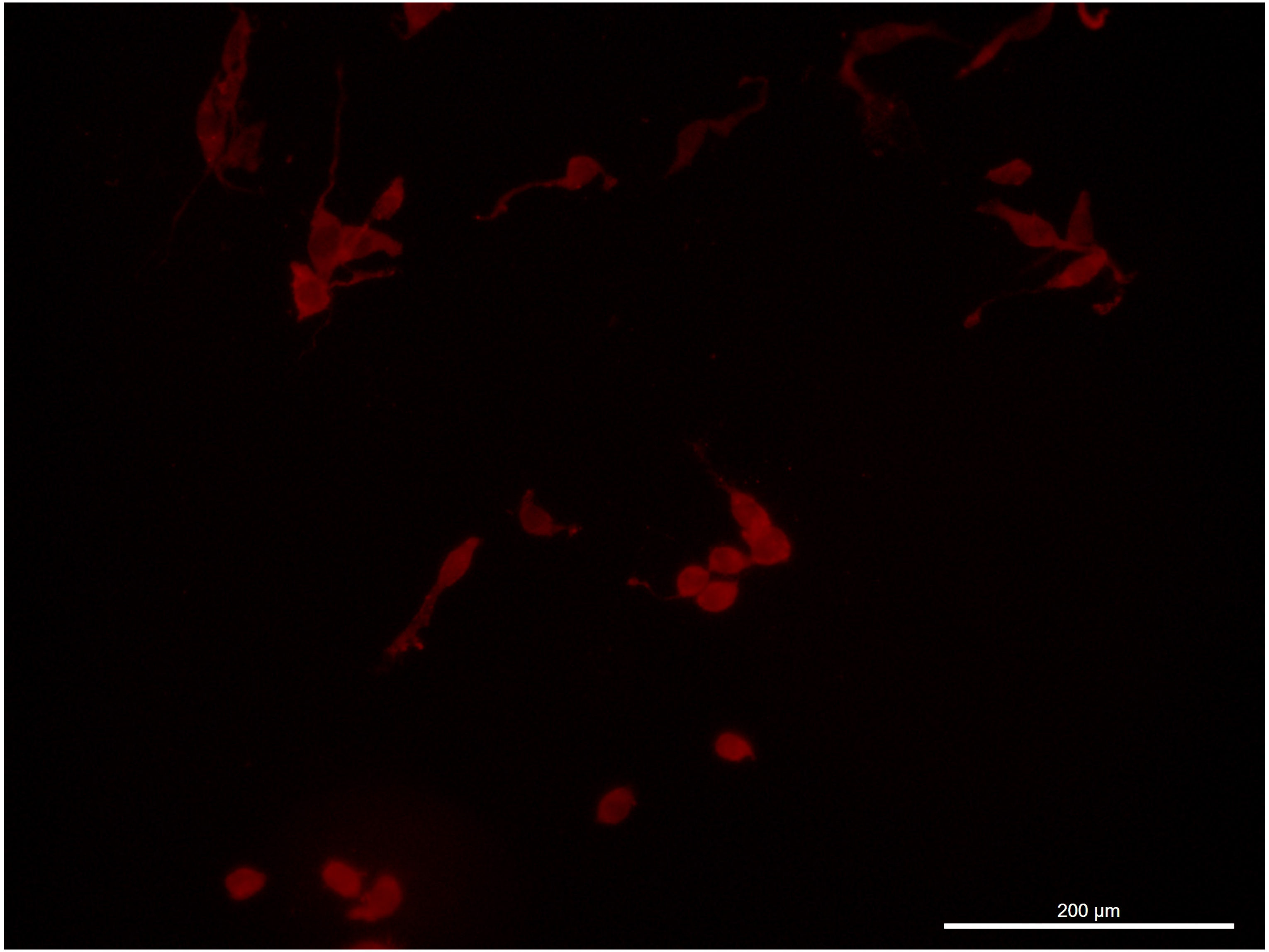

OE-DAPI

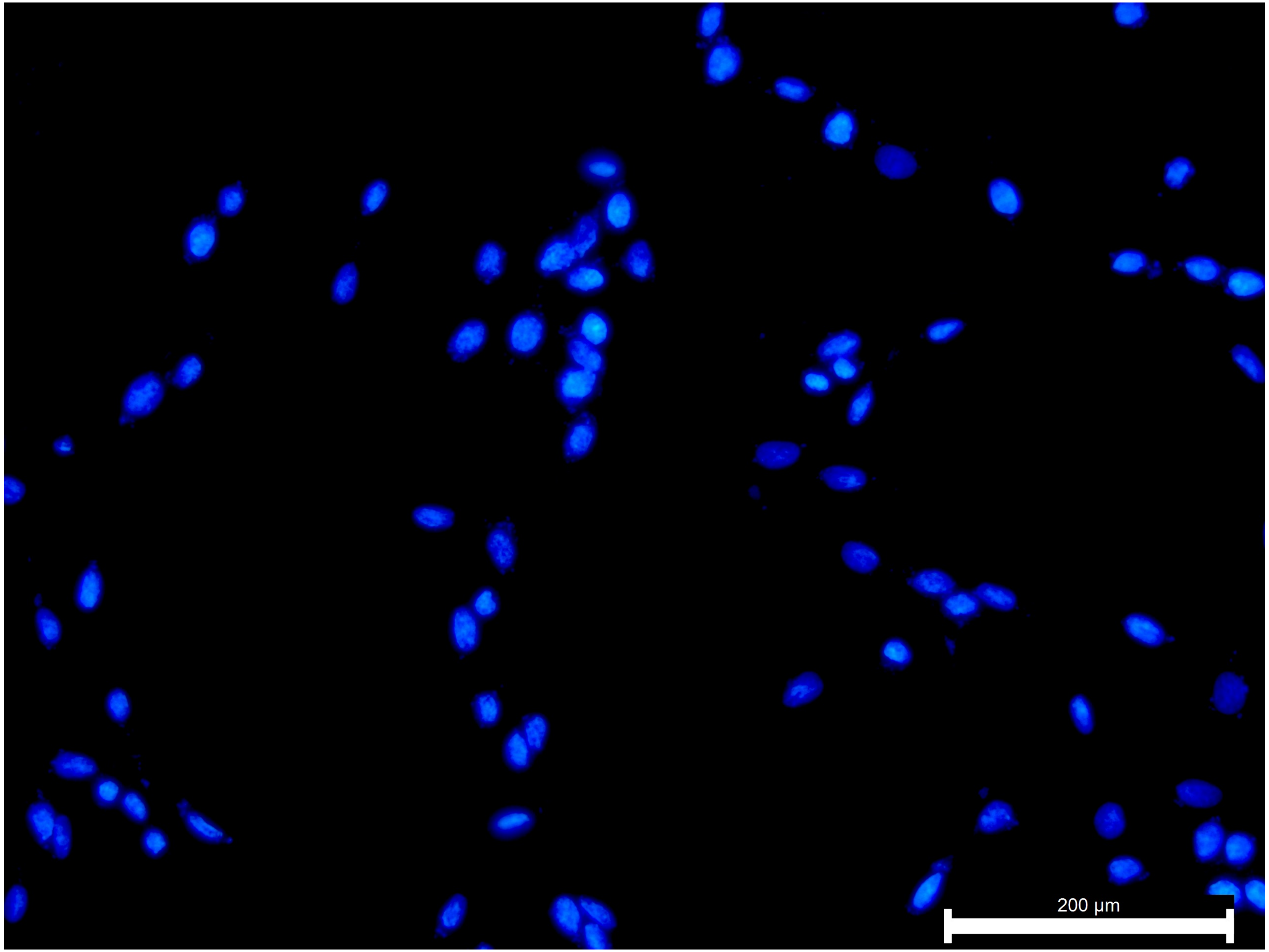

OE-merge

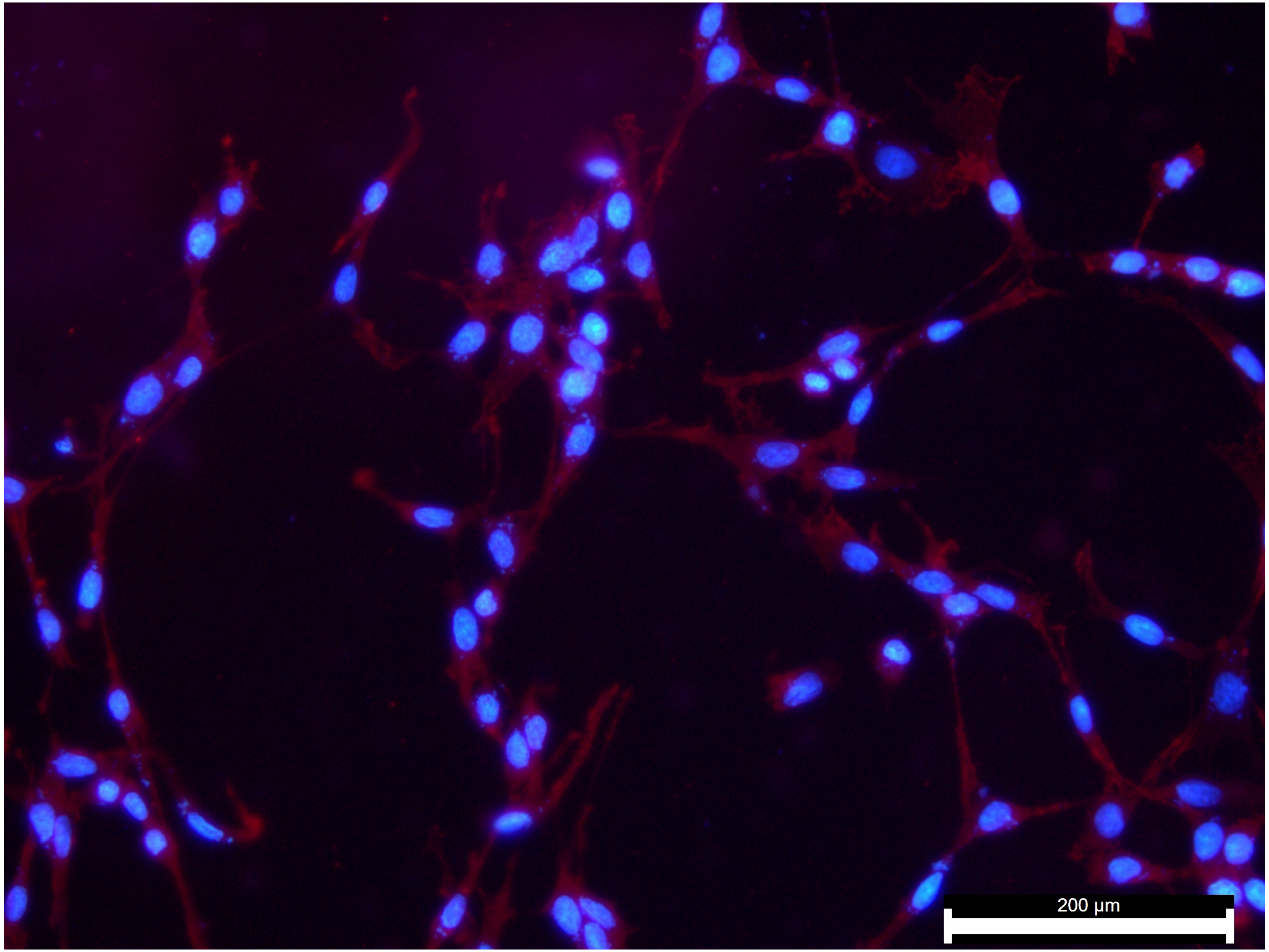

Supplement: Supplementary file 2 [file Data_Sheet_2.PDF]
